# Reliance on blue, green, and brown energy channels drives a shift in the trophic position of riparian spiders

**DOI:** 10.1002/ecy.70264

**Published:** 2026-01-14

**Authors:** Grégoire Saboret, Bastiaan J. W. Drost, Carmen Kowarik, Maja Ilić, Martin M. Gossner, Carsten J. Schubert

**Affiliations:** ^1^ Department of Surface Waters – Research and Management Eawag, Swiss Federal Institute of Aquatic Science and Technology Kastanienbaum Switzerland; ^2^ Department of Environmental Systems Science ETH Zürich Zürich Switzerland; ^3^ WSL (Swiss Federal Institute for Forest, Snow and Landscape Research), Forest Health and Biotic Interactions, Forest Entomology Birmensdorf Switzerland; ^4^ Department of Aquatic Ecology Eawag (Swiss Federal Institute of Aquatic Science and Technology) Dübendorf Switzerland; ^5^ Present address: Ocean Sciences Department, University of California, SantaCruz, Santa Cruz California USA; ^6^ Present address: University of Cologne, Institute for Zoology, General Ecology Cologne Germany

**Keywords:** amino acid isotopes, compound‐specific stable isotopes, food chain length, meta‐ecosystem, riparian predator, spider, trophic position

## Abstract

Understanding the mechanisms shaping food chain length (FCL) has long been central to food web ecology. FCL is a key determinant of stability, energy flow efficiency, and biodiversity maintenance, but there is an ongoing debate about its underlying drivers. It is particularly important in meta‐ecosystems, where predator trophic position (TP) is influenced by multiple energy channels. In this study, we focused on spiders in riparian ecosystems, which rely on resources linked to distinct energy channels: blue (algal herbivory), green (terrestrial herbivory), and brown (terrestrial detritivory). We applied nitrogen isotope analysis of amino acids to estimate the TP of both spiders and their prey. This method is a powerful tool for determining TP from a single sample and even allows for capturing decomposer trophic steps. However, the TP estimate requires special care for riparian spiders, as spiders show a specific trophic discrimination factor (TDF_Glx‐Phe_), and that energy channel use can confound the TP estimate. Our detailed food web resolution supports the use of specific parameters for spiders, particularly the low trophic discrimination factor (TDF_Glx‐Phe_ ~ 2‰), and raises caution about the importance of estimating resource use of predators to estimate their TP. We show that the primary factor driving variation in spider TP is the energy channel they utilize, from blue (TP ~ 2.9) to green (TP ~ 3.6) to brown (TP ~ 4.1). This increase was largely due to prey omnivory in green channels, and microbial and fungal decomposers serving as an initial trophic step between litter and invertebrate detritivores in brown channels. We propose that this pattern is likely influenced by differences in basal nutritional quality, which increases from brown (low) to green (medium) and to blue (high) sources. This suggests that shifts in energy channels within meta‐ecosystems in the course of global change (e.g., climate warming, eutrophication and land‐use change) may significantly impact FCL, with significant consequences for trophic interactions, nutrient fluxes, and biomagnification processes.

## INTRODUCTION

Food chain length (FCL), defined as the number of trophic steps between primary producers and top predators, is a key indicator of ecosystem functioning (Pimm & Lawton, 1977), yet the mechanisms that determine FCL remain debated (Post et al., 2000; Potapov, Brose, et al., 2019; Stukel et al., 2024). Although largely interconnected (Gounand et al., 2018), different ecosystems, such as aquatic and terrestrial, are traditionally studied in isolation. In these transition zones, aquatic (“blue”) and terrestrial, which is further divided into herbivory‐based (“green”) and detritivory‐based (“brown”) energy channels, come together. In blue ecosystems, hereafter defined as relying on algae, food webs are typically size‐structured, with energy limitations being a main driver of FCL (Décima, 2022; Takimoto & Post, 2013; Ziegler et al., 2015), and context‐specific factors such as environmental stability playing a role (Sabo et al., 2010). In contrast, terrestrial ecosystems feature large plant biomass that is defended against consumers by structural compounds, leading to the development of non‐size‐structured food webs (Shurin et al., 2006). Here, organisms of various sizes have evolved specialized strategies to exploit green resources, resulting in more complex drivers of FCL (Potapov et al., 2021; Young et al., 2013). The brown channel, where bacteria and fungi release energy from recalcitrant plant matter, is increasingly recognized to play a critical role in elongating FCL in terrestrial ecosystems (Manlick et al., 2023; Pauli et al., 2019; Steffan et al., 2019). While many theories and empirical observations address FCL, most studies focus on a single ecosystem type, while few explore how FCL is affected by energy channel use in meta‐ecosystems, that is, ecosystems that exchange energy, nutrients and organisms (Loreau et al., 2003). In a changing world, where food webs are being rewired (Bartley et al., 2019), understanding the connection between FCL and predator resource use is crucial for mapping the flow of energy and organic matter in meta‐ecosystems.

Riparian ecosystems, which exist at the interface between rivers or streams (blue) and terrestrial habitats (green and brown), represent common and important meta‐ecosystems (Gregory et al., 1991). These zones provide critical habitats for species with both aquatic and terrestrial life stages, including many insects and amphibians. Terrestrial consumers in riparian areas often benefit from blue resources (Twining et al., 2025). For instance, blue prey is rich in essential biomolecules like long‐chain polyunsaturated fatty acids (PUFAs) (Kowarik et al., 2021; Twining et al., 2018). Global environmental change, such as land‐use change and intensification, directly affects terrestrial ecosystems (Hao et al., 2025), but also aquatic ones (Ohler et al., 2024; Price et al., 2019). These effects are amplified, but they impact terrestrial and aquatic food webs differently (Ho et al., 2022), making riparian ecosystems particularly vulnerable (Capon et al., 2013; Stella & Bendix, 2019). Among riparian predators, spiders stand out as key subjects of study due to their ubiquity and role as essential trophic links between primary consumer invertebrates and higher‐level consumers (Bucher et al., 2015; Collier et al., 2002; Schmitz & Suttle, 2001). In particular, spiders can exploit prey of the blue, green, and brown energy channels (Bliska et al., 2024; Kowarik et al., 2021; Saboret, Drost, et al., 2024). Understanding how spiders' energy channel use relates to their trophic position (TP) is crucial to unravel how energy and organic matter are transferred to riparian predators, and contributes to our understanding of the drivers of FCL in meta‐ecosystems.

Over the past two decades, compound‐specific stable isotope analysis of amino acids (CSI‐AA) has overcome many limitations in understanding trophic relationships in ecosystems (Whiteman et al., 2019). One key advantage of separating amino acids (AAs) is the ability to distinguish between source AAs, such as phenylalanine (Phe), which exhibit minimal ^15^N fractionation along food chains, and trophic AAs, such as glutamic acid (Glu), which show significant trophic discrimination (Chikaraishi et al., 2009; McClelland & Montoya, 2002; Ohkouchi et al., 2017). Consequently, the TP of a consumer can be directly estimated from the difference in δ^15^N values between trophic and source AAs, providing TP with high resolution (Chikaraishi et al., 2014). The substantial ^15^N fractionation in Glu (~7–8‰) occurs due to AA deamination, where Glu plays a central role in metabolism. As a result, the TDF, which describes ^15^N enrichment of Glu between a consumer and its prey, is globally conserved across taxa and habitats (O'Connell, 2017). Remarkably, even heterotrophic bacteria and fungi exhibit similar TDF (Steffan et al., 2015, 2017), allowing this method to detect decomposer trophic steps that are traditionally difficult to assess (Manlick et al., 2023). However, the TDF of Glu may vary with food source quality, such as protein content, and consumer metabolism (Karlson et al., 2024; Whiteman et al., 2019). In particular, nitrogen excretion modes have been proposed as major drivers of TDF variation (Germain et al., 2013; McMahon & McCarthy, 2016). While CSI‐AA seems particularly promising to determine the TP of spiders, the only feeding trial has revealed unusually low TDF for spiders (Pollierer et al., 2019). Thus, the broader applicability of CSI‐AA remains uncertain as spiders require the use of a specific TDF.

In this study, we leverage CSI‐AA to link resource use from blue, green, and brown energy channels to the TP of consumers, focusing on spiders as key predators within riparian ecosystems. Our work builds upon a previous study that assessed resource use in invertebrates by analyzing δ^13^C of essential AAs and PUFA abundance (Saboret, Drost, et al., 2024). First, we assessed whether our data were compatible with spider‐specific TDF by comparing δ^15^N_AA_ values of spiders and their prey with the lowest TPs. Second, we determined the TP of both spiders and their prey to understand differences in FCL between blue, green, and brown energy channels.

## METHODS

### Field site and samples

We collected samples at the Necker, a 6th‐order stream in northeast Switzerland (47.39281 N, 9.09015 E, Appendix S1: Figure S1), in June 2020 and May 2023. We collected spiders by hand, visually screening the litter and vertical structures, without size and species distinction, including ground‐dwelling spiders and web‐building spiders, and from the shoreline up to a distance of 100 m from the stream. By sampling spiders with distinct hunting strategies and varying distances from the stream, we aimed to capture the diversity of energy channel use (Kowarik et al., 2021, 2023). In 2020, we also collected stream invertebrates via kick sampling, including mayfly and caddisfly larvae (blue sources), and terrestrial invertebrates using sweep net sampling, such as plant bugs and leaf beetles, which are major herbivores (green sources). In 2023, we manually collected detritivorous invertebrates (brown sources), including key taxa such as ground beetles, springtails, and mites, using a sifting net and tweezers. Our sampling, therefore, covered the energy channels derived from algae (blue), leaf litter (brown), and aboveground vegetation (green). All samples were transported to the laboratory, identified to the lowest practicable taxonomic level, and stored at −20°C until further analysis. No specific permits were required for sampling, and all fieldwork was conducted in accordance with Swiss guidelines and regulations.

### Sample classification

We classified potential prey into three habitat‐based groups from where they were caught: blue (aquatic), brown (soil and leaf litter), and green (grass, shrubs and trees). We assigned taxa to coarse trophic levels (herbivore, detritivore, omnivore, and predator), based on the literature. We assumed that herbivores and detritivores integrated the energy channels of their respective habitats (Post, 2002; Potapov et al., 2021), and used them as mixing model source end‐members (Phillips & Gregg, 2003). Energy channel use was therefore a continuous metric and could differ from the habitat classification (see *Methods: Energy channel use*). Most of the collected spiders belonged to two families: Lycosidae (ground‐dwelling spiders) and Tetragnathidae (web‐building spiders). Due to their abundance, we focused the analysis on these two groups, hereafter referred to simply as ground spiders and web spiders, respectively. More information on the samples can be found in Saboret (2025) (see https://osf.io/5e6g8/files/, Invertebrate_AA_isotope_data.xlsx).

### Amino acid isotope analysis

We performed CSI‐AA in 44 samples, of which we measured fatty acids and δ^13^C_AA_, as reported in Saboret, Drost, et al. (2024), and δ^15^N_AA_ (this study). In brief, we weighed 2–10 mg of dry weight, and pooled samples when necessary (e.g., CSI‐AA in springtails required >50 individuals each). For CSI‐AA δ^15^N, we hydrolysed the pellets after fatty acid extraction and subsequently derivatized the free AAs to N‐Acetyl Methyl Ester (NACME‐AA) derivatives following previous protocols (Corr et al., 2007; Larsen et al., 2013). More details can be found in Saboret, Drost, et al. (2024).

We determined the δ^15^N of AAs using a Delta V+ Advantage (Thermo) coupled to a combustion tube. We prepared an in‐house AA standard containing 15 AAs of interest, in order of elution: alanine (Ala), glycine (Gly), valine (Val), leucine (Leu), isoleucine (Ile), proline (Pro), aspartic acid (Asp), serine (Ser), threonine (Thr), Glu, methionine (Met), Phe, lysine (Lys), tyrosine (Tyr), histidine (His). We purchased individual AAs through Sigma‐Aldrich (purity >99.5%) and determined their isotopic composition using five replicates on EA‐IRMS. We ran the AA standard alongside the samples at the beginning, at the end, and every five samples of each sequence. Before and after each AA standard, we ran an external caffeine standard of known isotopic composition (Schimmelmann, University of Indiana).

Because during hydrolysis glutamine (Gln) and asparagine (Asn) are deaminated to Glu and Asp, respectively, we reported measurements for Glx (Glu and Gln) and Asx (Asp and Asn). This means that δ^15^N_Glx_ corresponds to the δ^15^N of the amine group of the α‐carbon of Glu and Gln. We reported the values for 10 AAs for which we achieved sufficient peak separation and intensity: Ala, Asx, Glx, Ile, Leu, Phe, Pro, Ser, Thr and Val.

We calculated the SD based on triplicate measurements. The SD of the in‐house AA standard was 1.1‰ (*N* = 44, ranging from 0.6‰ for Ala to 1.3‰ for Pro). The between‐samples SD of the internal standard norleucine was 0.7‰. We conducted three batches of derivatization, whose AA standard of the reported AA did not differ significantly (ANOVA tests, *p* > 0.05). We calculated for each individual measurement the difference between δ^15^N_Glx_ and δ^15^N_Phe_, noted as Δ^15^N_Glx‐Phe_. The AA standard Δ^15^N_Glx‐Phe_ deviated on average by 0.5‰ from known Δ^15^N_Glx‐Phe_, with a SD of 0.8 ‰. Because of within‐run measurement covariance, this SD (σ_ΔGlx‐Phe_) was less than the only propagation of uncertainty given by σ_15NGlx_ − σ_15NPhe_ = 1.1‰. We classified AAs as source (Phe), metabolic (Thr), other (Ser) and trophic (Ala, Asx, Glx, Ile, Leu, Pro, Val) (Pollierer et al., 2019; Whiteman et al., 2019). We calculated the mean trophic δ^15^N_AA_ as the mean δ^15^N of trophic AAs.

### Energy channel use

To explore the relationship between trophic structure and energy channels (blue, green, and brown), we estimated the energy channel use for each sample. Accurately determining the source reliance is crucial for estimating TP using the Δ^15^N_Glx‐Phe_ method, as algae and vascular plants exhibit significant differences in ^15^N fractionation between Glx and Phe (see *Methods: Trophic position*). We defined the blue energy channel as relying on aquatic autotrophs, i.e., algae. However, an important question is whether the blue herbivores could rely on allochthonous organic matter, particularly in the form of leaf litter (Brett et al., 2017). Several biomarkers indicated a dominant reliance on algal (autochthonous) resources by emerging invertebrates at our study site. First, the bulk (Kowarik et al., 2021), and the mean essential amino acid (EAA) δ^13^C values were highly depleted (Appendix S1: Figure S2a), which is characteristic of stream algae (Finlay, 2004). Second, invertebrate PUFA profiles were typical of algal consumers (Appendix S1: Figure S2b), with high levels of EPA and especially a high omega‐3/omega‐6 ratio (mean = 3, range = 1–6), indicative of a strong algal contribution to their diet (Guo et al., 2016; Torres‐Ruiz et al., 2007). The δ^13^C of PUFAs was also depleted, further supporting an autochthonous origin (Kowarik et al., 2021). Additionally, different phyla of primary producers leave distinct δ^13^C offsets in EAAs that are preserved up the food chain, a method known as EAA fingerprinting (Larsen et al., 2009). In our case, emerging stream invertebrates matched algal fingerprints (Appendix S1: Figure S2c,d), particularly characterized by negative normalized Phe δ^13^C (Appendix S1: Figure S2e), a pattern typical of algae (Saboret et al., 2023). While each method on its own has limited quantitative resolution (as further discussed in Appendix S1: Section S2), all markers consistently point to a dominant autochthonous contribution (>90%) in emerging insects, especially mayflies, in line with previous studies (Bellamy et al., 2019; Cortés‐Guzmán et al., 2022; Ishikawa et al., 2020). Thus, biomarkers supported that the blue energy channel was indeed based on algae production, in line with our definition.

We estimated energy channel use of all invertebrates based on δ^13^C of a combined mixing model using herbivores and detritivores as source end‐members, based on four essential AAs and PUFA abundance (Appendix S1: Figure S3), following the approach outlined by Saboret, Drost, et al. (2024). Saboret, Drost, et al. (2024) demonstrated that these two approaches consistently describe dietary sources and, when used together in a mixing model, can reduce uncertainty in source reliance estimates. Thus, our continuous estimates of energy channel use for all consumers were derived from herbivores and detritivores, which we treated as dietary sources.

We categorized the samples into four groups based on energy channel use: blue, green, brown, and mixed. The blue, green, and brown groups consisted of samples that relied on a single source by more than 70%, as inferred from the mixing model. This energy channel grouping, based on the mixing model, was different from the habitat classification. In particular, one aquatic herbivore (stonefly) and one leaf litter detritivore (millipede) were shown to have green dependence (>30%), and therefore were classified in the mixed diet category. The three other groups were dominated by source specialists, with an average reliance of 89%–90% on their focal source. The mixed category included samples that did not have a dominant source (Appendix S1: Figure S4). Spiders were found across all energy channel categories, while other predators relied on the brown and mixed channels, except for two beetle samples (genus *Bembidion*), which relied on the blue channel.

We checked whether δ^15^N_AA_ values were consistent with energy channel use. To do so, we used Kruskal–Wallis tests to determine whether the δ^15^N of each AA (δ^15^N_AA_) differed between above‐described groups, followed by post hoc comparisons using Fisher's least significant difference with Holm–Bonferroni corrections from the *agricolae* package (De Mendiburu & Simon, 2015). We performed this analysis only for blue, green, and brown energy channels (not mixed ones as they are not informative of a specific channel). We conducted two analyses, one for the potential spider prey (only herbivores and detritivores), and one for spiders. We normalised δ^15^N of all AAs (except Phe) to δ^15^N_Phe_. This normalized δ^15^N_AAs_ is valuable because it excludes baseline differences and retains only differences in primary producer fractionation (i.e., β values) and trophic enrichment (i.e., different amounts of trophic steps and/or TDF).

### Pattern of ^15^N enrichment from basal prey to spiders

The ^15^N fractionation between a consumer and its diet, defined by the relative δ^15^N difference between the two, is a key parameter for accurately determining a consumer's TP. The only feeding experiment on spiders has revealed unusual ^15^N fractionation, associated with a low trophic discrimination factor of Glx over Phe (TDF_Glx‐Phe_) (Pollierer et al., 2019), which is the parameter used in the TP equation (see *Methods: Trophic position*).

Here, we explored whether the laboratory‐derived parameters are compatible with our field values. To do so, we estimated ^15^N enrichment between spiders and prey of the lowest TPs (i.e., herbivores or detritivores) across the three different energy channels, hereby referred to as “^15^N enrichment above basal”. These values represent the maximum ^15^N enrichment, meaning that significantly lower values than those from feeding trials would indicate either data incompatibility or issues with source determination.

We calculated SD using propagation of uncertainty between spiders and sources. To test for ^15^N enrichment differences, we performed pairwise Welch's *t*‐tests with Bonferroni corrections using the package BSDA (Arnholt & Evans, 2017). Specifically, we compared our data with reference values of TDF_Glx‐Phe_ for spiders (mean = 1.9, SD = 1.4, *n* = 23) and for terrestrial organisms with uric acid excretion (mean = 7.5, SD = 0.5, *n* = 52) (McMahon & McCarthy, 2016).

### Trophic position

We estimated TPs using the method from (Chikaraishi et al., 2009):

$$\mathrm{TP}=\left(\Delta^{15}N_{\mathrm{Glx}‐\mathrm{Phe}}-\beta_{\mathrm{mix}}\right)/{\mathrm{TDF}}_{\mathrm{Glx}‐\mathrm{Phe}}+1.$$

TDF_Glx‐Phe_ is the relative trophic enrichment of Glx over Phe for each consumer food step. This method has proven successful for many invertebrate taxa (Chikaraishi et al., 2014; Lux et al., 2024; Potapov, Tiunov, et al., 2019; Steffan et al., 2017, 2019), and even for micro‐organisms such as fungi and heterotrophic bacteria (Steffan et al., 2015). However, the only experimental study on arachnids showed that arachnids have an atypically low TDF_Glx‐Phe_ (Pollierer et al., 2019). Therefore, we estimated TP of spiders (TP_spiders_) by adding the spider trophic step into the equation, and subtracting their TDF, similar to Germain et al. (2013). In this equation, both primary producers (β_mix_) and spiders (TDF_Spider_) are known, resulting in TP being set to +2, while the additional contribution to TP comes from intermediate consumers, with the universal TDF (TDF_Glx‐Phe_) driving differences in measured Δ^15^N_Glx‐Phe_ (see illustration in Appendix S1, Figure S5):

$${\mathrm{TP}}_{\text{spiders}}=\left(\Delta^{15}N_{\mathrm{Glx}‐\mathrm{Phe}}-\beta_{\mathrm{mix}}-{\mathrm{TDF}}_{\text{spider}}\right)/{\mathrm{TDF}}_{\mathrm{Glx}‐\mathrm{Phe}}+2,$$

where β_mix_ is the relative trophic enrichment of Glx over Phe in primary producers. Because this value is different for algae (β_algae_) and C3 vascular plants (β_vascular_) (Chikaraishi et al., 2014), we used a mixed value, considering the relative contribution of one source or another, following Zhang et al. (2019):

$$\beta_{\mathrm{mix}}=\beta_{\text{vascular}}\times f+\beta_{\text{algae}}\times \left(1-f\right),$$

with *f* being the reliance originating from vascular plants (green and brown, see *Methods: Energy channel use*). Importantly, the energy channel use is based on essential AAs, and therefore reflects the source of proteins, and so no correction for macromolecular composition is needed.

The different parameters mean and SD values were taken from literature values (Table 1).

**TABLE 1:** Summary of trophic position (TP) estimate parameters.

Parameter	Mean	SD	Reference
TDF	7.64	0.6	Chikaraishi et al. (2009)
TDF_spiders_	1.89	1.39	Pollierer et al. (2019)
β_algae_	3.4	0.9	Chikaraishi et al. (2009)
β_vascular_	−8.4	1.6	Chikaraishi et al. (2009)

Abbreviation: TDF, trophic discrimination factor.

### TP uncertainty

To estimate uncertainty around TP estimates, we measured TP SD by accounting for the propagation of uncertainty using first‐order Taylor series expansion (Matthews et al., 2020) in the package *propagate* (Spiess et al., 2018). We accounted for uncertainty in parameters (Table 1), and in measurement uncertainty (σ_ΔGlx‐Phe_), and run for each individual 10.000 Monte Carlo simulations. To characterize the sources of uncertainties, we fitted multiple linear regression of TP SD to the potential drivers of individual TP uncertainty, including TP, blue reliance, blue reliance uncertainty (SD), and analytical measurement precision (σ_ΔGlx‐Phe_). We selected explanatory variables using the corrected Akaike information criterion (AIC_c_) (Burnham et al., 2002), using the function *dredge()* in the MuMIn package (Barton, 2009).

### Differences in consumer TP between blue, green, and brown energy channels

Our main goal was to understand the link between energy channels (blue, green, and brown) and FCL in riparian ecosystems, using spider TP as a proxy. To do so, we used Kruskal–Wallis tests to compare TP between dietary categories for both spiders and their prey, followed by post hoc comparisons using Fisher's least significant difference test and Holm–Bonferroni corrections. Secondly, to test the relationship between energy channel use and spider TP, we fitted a multiple linear regression. In essence, we used linear equations to fit a mixing model of TP, assuming that each energy channel is associated with a value of TP. We visually checked linearity, homogeneity of variance and normality of residuals.

## RESULTS

### Linking δ^15^N_AA_ to the different energy channels

**FIGURE 1:**
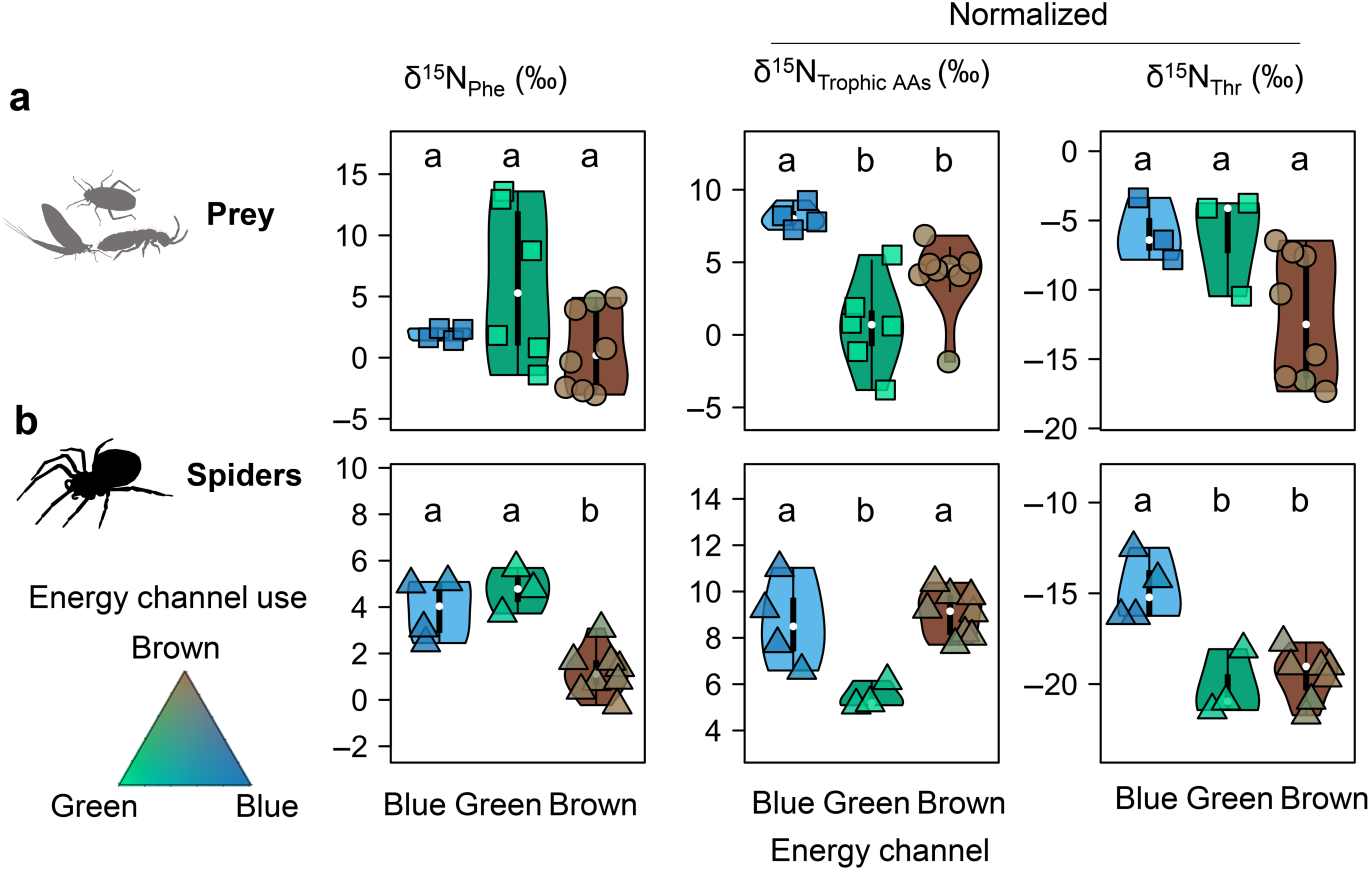
Pattern of δ^15^N_AAs_ in the different energy channels. Violin plots (left to right) showing source (Phe), trophic (mean values), and metabolic (Thr) δ^15^N_AA_ in the three energy channels. The top panel (a) represents prey (square and circle show herbivore and detritivore, respectively), and the bottom panel (b) represents spiders. Letters indicate significant differences between groups (*p* < 0.05, Kruskal–Wallis test with post hoc comparisons using the Fisher's least significant difference with Holm–Bonferroni corrections). Figure created with species silhouettes sourced from Phylopic.org under a CC0 1.0 Universal Public Domain Dedication license.

**FIGURE 2:**
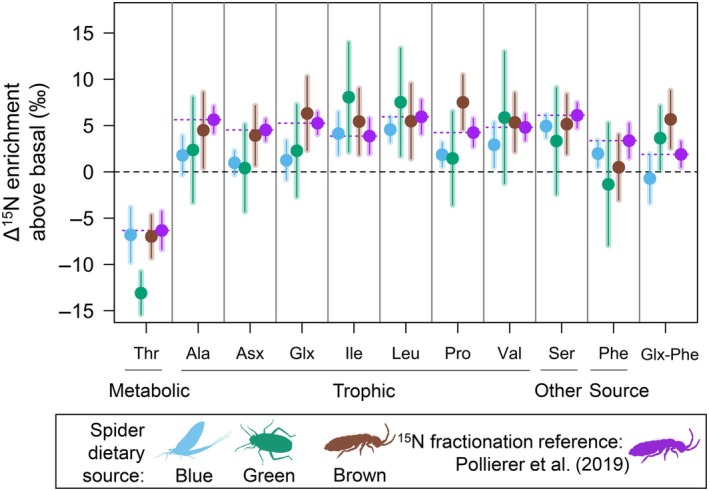
Pattern of amino acid ^15^N enrichment above basal between spider and their prey of the lowest trophic positions. Comparison of the ^15^N enrichment between spiders and prey of the lowest trophic positions (herbivores and detritivores) for the three different energy channels, with reference values from a feeding experiment (Pollierer et al., 2019). Dotted line shows the reference value, circles indicate the mean, and error bars represent mean ± 1 SD. There were no significant differences between groups (*p* < 0.05, Welch's *t*‐test with Bonferroni corrections) for any of the amino acids (AAs). AAs are classified following their N metabolism. Glx‐Phe represents the trophic discrimination factor (TDF) used for trophic position estimates. Figure created with species silhouettes sourced from Phylopic.org under a CC0 1.0 Universal Public Domain Dedication license.

The three sources (i.e., spider prey) did not show significant differences in δ^15^N_Phe_ (Figure 1a). Brown prey exhibited the lowest δ^15^N_Phe_ (−3‰ for springtails, Collembola, Figure 2), and green prey displayed a very large variation, from −1‰ (plant bugs, *Harpocera thoracica*) to 14‰ (leaf beetles, *Oulema* sp.). This variability likely reflects the variability of N metabolism in terrestrial ecosystems (see *Discussion*). The high variability in δ^15^N_Phe_ was not observed in spiders, where δ^15^N_Phe_ ranged between 0% and 6‰. However, spiders relying on brown sources had significantly lower δ^15^N_Phe_ (Figure 1b).

Blue herbivores showed significantly higher normalized δ^15^N (i.e., relative to δ^15^N_Phe_) in mean trophic AAs than green herbivores and detritivores (Figure 1a), and for all trophic AAs, except for Leu and Ile, where differences to brown sources were not significant (Appendix S1: Figure S6). Spiders feeding on green sources showed significantly lower normalized δ^15^N values for Thr, and spiders feeding on blue sources showed significantly higher normalized δ^15^N values for trophic AAs, compared to those relying on other sources (Figure 1b). Thus, each feeding source was associated with a significantly different pattern, supporting the distinctiveness of the three energy channels. All supporting data are provided in Appendix S1: Figure S7.

### Patterns of AA ^15^N enrichment above basal

The ^15^N enrichment above basal (i.e., ^15^N enrichment from prey of the lowest TPs to spider) was not significantly different between sources for all AAs (Figure 2). Spiders relying on green sources had high uncertainty in ^15^N enrichment above basal due to large variability in δ^15^N_AA_ from potential prey (Figure 2). We found that the ^15^N enrichment above basal for Phe was centered around 0.4‰, and not significantly different from zero, supporting our estimates of food source reliance. The values were, in general, slightly lower (Ala, Asx, Ser), close (Leu, Val), slightly higher (Ile), or variably different (Thr, Glx, Pro) than reference values from Pollierer et al. (2019), but not significantly different. This supported the compatibility between laboratory data and our inferences of food sources and prey assumptions. Higher ^15^N enrichment above basal than reference values was expected if spiders were not feeding on prey the lowest in the food chain (i.e., more trophic links). This could be the case for brown sources, which overall showed higher ^15^N enrichment in most AAs. We found that the maximum possible TDF_Glx‐Phe_, (assuming spiders feed on prey of the lowest TP) was low, especially in the blue energy channel (−0.8‰). Our average maximum TDF_Glx‐Phe_ was 2.9‰ (SD = 5.3‰), which was not significantly different from spider reference value (1.8‰, *p* = 0.48), but significantly lower than terrestrial insect reference value (7.5‰, *p* = 0.005). Overall, our data were consistent with feeding trial value for all AAs, and incompatible with universal TDF_Glx‐Phe_. (unless assuming a very high degree of spider herbivory). This supported our use of a specific TDF value for spiders.

### TP estimates across energy channels

**FIGURE 3:**
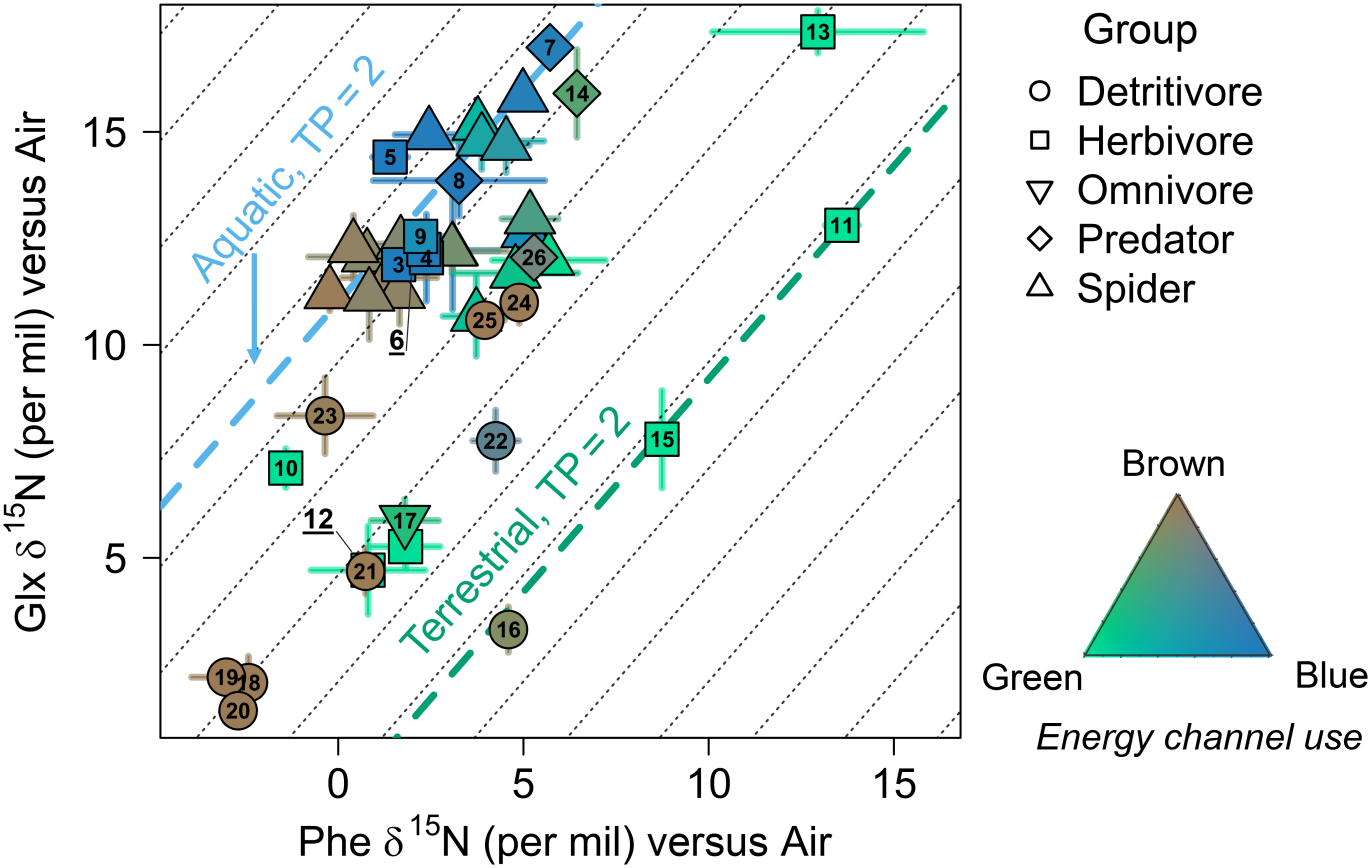
δ^15^N of Glx and phenylalanine (Phe) used for trophic position (TP) estimates. Each data point represents a sample, with shape indicating the coarse trophic group (with spiders being a separate group), and color reflecting energy channel use. Error bars represent one SD (analytical error). Dotted lines indicate trophic isoclines, while green and blue lines show the trophic isoclines for green and blue primary consumers (TP = 2), respectively. Numbers refer to sample taxon identification (except for spiders, represented by triangles), with references in Saboret (2025) (see https://osf.io/5e6g8/files/, Invertebrate_AA_isotope_data.xlsx) and Figure 4. Figure created with species silhouettes sourced from Phylopic.org under a CC0 1.0 Universal Public Domain Dedication license.

We used δ^15^N_Glx_ and δ^15^N_Phe_ to estimate the TPs of all invertebrates (Figure 3). Most of the variation in TP uncertainty was explained by the additive effect of TP (propagation of TDF uncertainty along the food chain), blue reliance uncertainty (i.e., SD around the estimate), blue reliance, and analytical precision (*R* ^2^ = 0.95, model weight >98%, Appendix S1: Figure S8). TP uncertainty was primarily influenced by the TP value itself, doubling as TP increased from 2 to 4 (Appendix S1: Figure S8a). Uncertainty in energy channel use also had a strong effect, with TP uncertainty increasing by 50% as the uncertainty in blue reliance rose from 0% to 25% (Appendix S1: Figure S8b).

**FIGURE 4:**
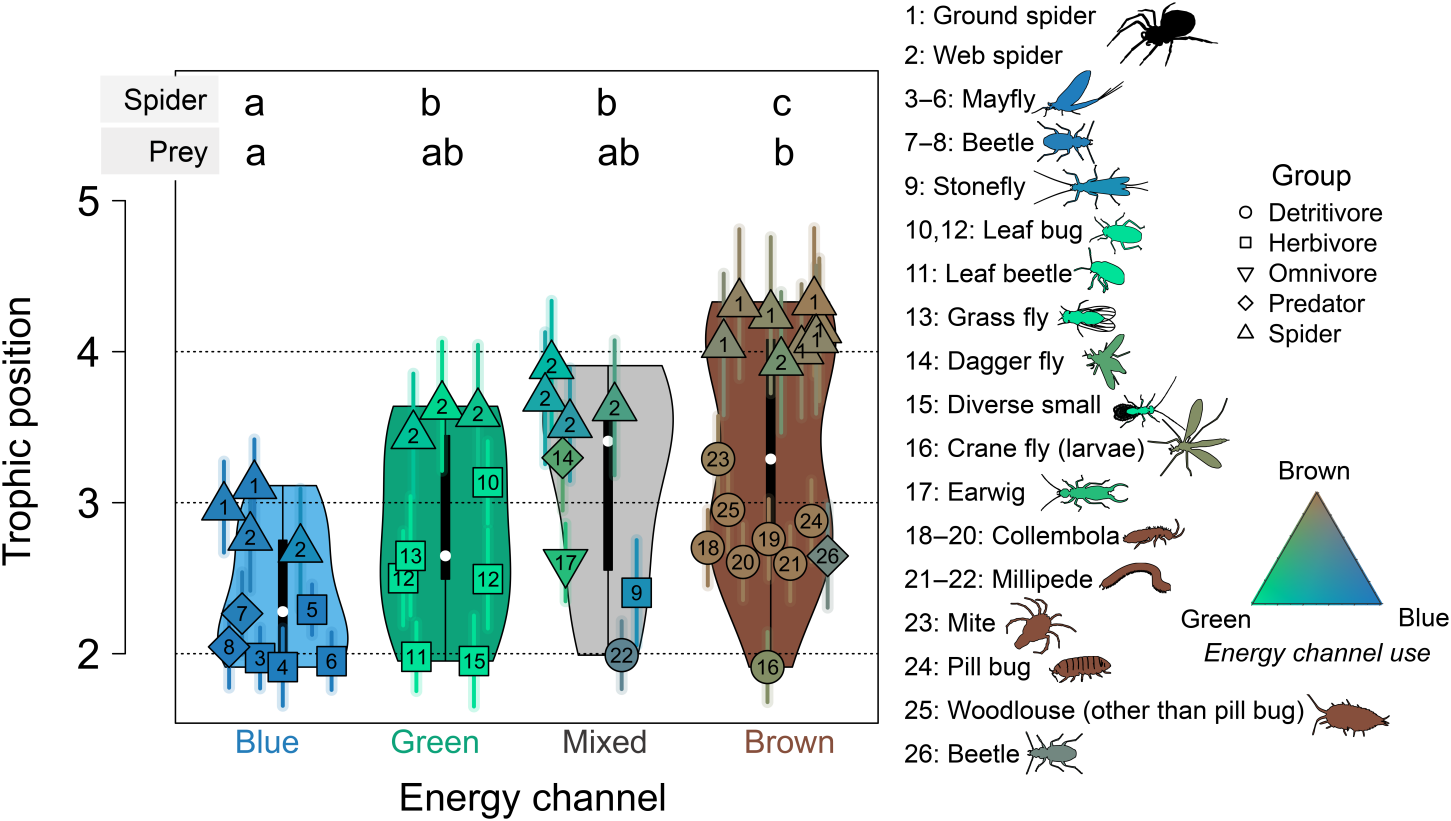
Trophic position estimates. Violin plots of trophic position (TP) for the energy channels, including mixed one (where all source reliances are <70%). Datapoints represent individual samples, with shape and color indicating grouping and energy channel use (as shown in Figure 3). Numbers correspond to coarse species classifications in Saboret, 2025) (see https://osf.io/5e6g8/files/, Invertebrate_AA_isotope_data.xlsx for more details). Error bars represent one SD, including the propagation of uncertainty. Letters indicate significant differences between spiders (*p* < 0.05, Kruskal–Wallis test with post hoc comparisons using the Fisher's least significant difference with Holm–Bonferroni corrections). Figure created with species silhouettes sourced from Phylopic.org under a CC0 1.0 Universal Public Domain Dedication license.

The TPs of invertebrates ranged from 1.9 (mayfly) to 4.3 (spider) (Figure 4). All four energy channels (blue, green, brown, and mixed) included at least one sample with a TP around 2, indicative of strict primary consumers. Blue invertebrates, primarily classified as herbivores (e.g., mayflies), had an average TP of 2.0. This was not significantly different from the average TP of green herbivores (mean = 2.5, *p* > 0.05), although some green herbivores, such as grass flies (*Geomyza* sp.) and leaf bugs (*Harpocera thoracica* and *Leptopterna ferrugata*), displayed elevated TPs between 2.5 and 3.1. Brown detritivores exhibited significantly higher TPs than blue herbivores (mean = 2.7, *p* < 0.05). Notably, common detritivores such as springtails and woodlice showed TPs ranging from 2.6 to 3.0, respectively.

**FIGURE 5:**
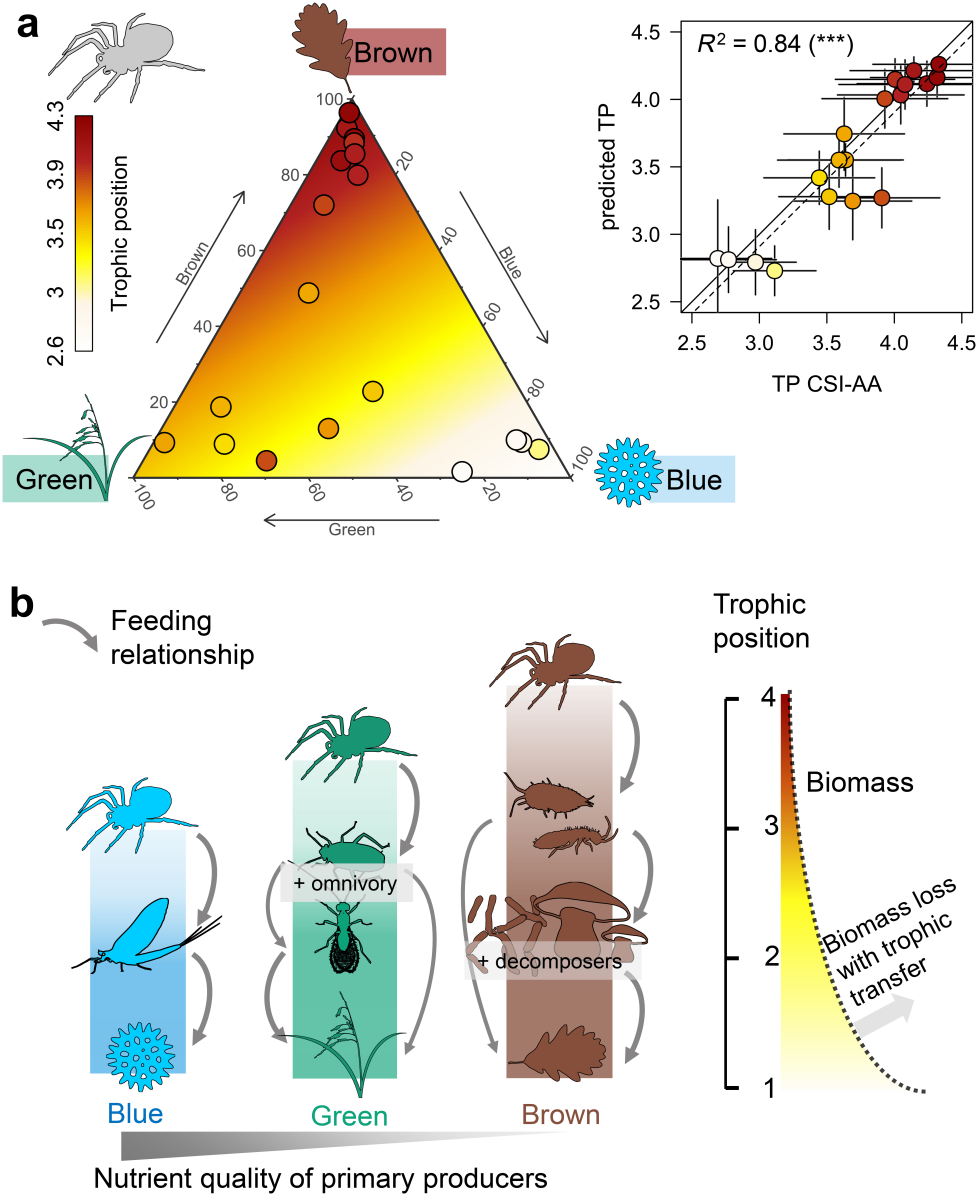
Link between energy channel and spider trophic position. (a) Ternary diagram showing the link between spider trophic position (TP, see color) and reliance on blue, green, and brown sources (axis). Symbol color represents the spider's TP, while the background color indicates the expected average TP for different energy channel mixtures, as inferred from the linear model. To the top‐right: Relationship between measured TP from the amino acid (AA) method (*x*‐axis) and estimated from source reliance (*y*‐axis). The full and dotted lines show the one‐to‐one and regression lines, respectively. (b) Conceptual diagram showing the food chains from blue, green, and brown sources, the expected nutrient quality of primary sources, and the flow of biomass up food chains. Figure created with species silhouettes sourced from Phylopic.org under a CC0 1.0 Universal Public Domain Dedication license.

Spiders showed a similar trend, with significant differences in TP between blue (mean 2.9), green, and mixed (mean 3.6 and 3.7, respectively), and brown (mean 4.1) energy channels (Figure 4). Spider TP could be explained by multiple linear regression with source reliance (linear model, *p* < 0.001, Appendix S1: Figure S9). Using this equation, we explicitly calculated TP from energy channel use (Figure 5a). This estimate closely fitted empirical values (*R* ^2^ = 0.84), with some outliers showing higher TP than estimated from their energy channel use alone (Figure 5a).

## DISCUSSION

### General findings

Our findings provide new insights into the TPs of riparian spiders and their prey, based on the strength of compound‐specific stable isotope analysis of amino acids (CSI‐AA). First, our results emphasize that estimating consumer TP in riparian ecosystems requires particular attention due to source estimate uncertainties. Second, they raise caution about the universality of CSI‐AA to estimate TP due to the unusually low TDFs of spiders. Third, our study demonstrates that reliance on different energy sources can influence the TP of predators, such as spiders, linking energy channels to FCL. Fourth, the results suggest that this relationship likely stems from intrinsic differences in the quality of primary producers fueling each energy channel (Figure 5b). Ultimately, our findings improve our understanding of how shifts in energy flow, driven by global change, may impact FCL, which, in turn, has implications for ecosystem functioning and resilience.

### Determining source reliance is important for accurately assessing the TP of riparian predators

In riparian ecosystems, where consumers rely on a mixture of blue (algae), green (plants), and brown (decomposing plants) energy channels, the interpretation of traditional bulk isotope analyses faces challenges in accurately estimating source reliance and consequently TP, due to source overlap and seasonal variations (Bliska et al., 2024; Bollinger et al., 2023; Kowarik et al., 2023; Siebers et al., 2021). One strength of CSI‐AA lies in its ability to assess source reliance, coupled with precise TP estimates based on the relative ^15^N enrichment in trophic versus source AAs (Chikaraishi et al., 2014). However, since different primary producers, such as algae and plants, have distinctly different baseline β values (i.e., ^15^N enrichment in trophic vs. source AA), TP estimates are highly sensitive to source reliance (Larsen et al., 2022). Ramirez et al. (2021) have previously noted that TP uncertainty can be substantial in top predators, as uncertainties in TDF and β values propagate across trophic levels. In our study, we emphasize the additive effect of source reliance, where TP uncertainty increases by 50% (one σ) as blue‐source reliance uncertainty rises from 0% to 25%. This challenge is not limited to our study but is likely widespread in ecological research, as top predators often couple energy from multiple primary producers, including C3 and C4 plants, as well as algae (Arsenault et al., 2022; Thorp & Bowes, 2017), which all exhibit distinct β values (Ramirez et al., 2021).

To improve estimates of source reliance, δ^15^N_AA_ analysis can provide additional insights with Phe serving as a useful indicator of baseline values (Harada et al., 2022; Ishikawa et al., 2014). In our study, we found that spiders relying on brown sources had an average δ^15^N_Phe_ value that was ~3‰ lower than spiders relying on blue or green sources. This lower δ^15^N_Phe_ aligns with the low values that we observed in springtails (−3‰), which may result from the preferential assimilation of a ^15^N‐depleted Phe pool from detritivores in leaf litter (Pollierer et al., 2019). Thus, δ^15^N_Phe_ could help distinguish between fresh (blue, green) and brown energy channels, although it was also reported that decomposer springtail δ^15^N_Phe_ is additionally influenced by leaf litter composition (Li et al., 2022; Lux et al., 2024). We also observed a broad range of δ^15^N_Phe_ variation in invertebrates from the green energy channel. Herbivores, such as most beetles, typically show little or negative ^15^N fractionation in Phe (Steffan et al., 2017; Takizawa et al., 2020). Thus, this variability likely reflects differing dietary sources. For example, leaf‐chewing beetles specialized on Poaceae (e.g., *Oulema* sp.) showed high δ^15^N_Phe_ values (up to 14‰), while plant bugs (*Harpocera thoracica*), specialized on sucking on oak reproductive organs, exhibited the lowest values (−1‰). Such differences are likely driven by variations in nitrogen acquisition strategies and metabolism of different plant species (Ramirez et al., 2021). In addition, reproductive organs can exhibit significant ^15^N‐depletion in Phe compared to leaves (Takizawa et al., 2017), potentially explaining the low δ^15^N_Phe_ values observed in plant bugs feeding on oak reproductive organs. Overall, the wide variability in δ^15^N_Phe_ among green herbivores limited its utility in distinguishing the aquatic versus terrestrial reliance of spiders.

In contrast, the relative ^15^N enrichment in trophic AAs was lower for spiders relying on green sources, which directly resulted from the β value of green producers (Zhang et al., 2019). Such a pattern has been suggested to be used for inferring aquatic‐terrestrial reliance in herbivores (García‐Seoane et al., 2023). However, we raise caution with this method in predators, such as spiders, because: (1) the reliance estimate heavily depends on accurate TDFs, which is generally not the case (see below); (2) trophic AAs tend to be collinear so they do not separate sources well; and (3) TP can act as a confounding factor, as illustrated by the fact that spiders relying on both blue and brown sources exhibited similar δ^15^N of trophic AAs.

### Spiders are an almost invisible trophic step in the food chain when using δ^15^N of Glu

Directly estimating spider TP can be challenging due to high uncertainty in bulk isotope fractionation (Oelbermann & Scheu, 2002). CSI‐AA has significantly advanced the field of food web ecology by enabling the distinction between source and trophic amino acids, in particular Phe and Glx, respectively (Chikaraishi et al., 2009; McClelland & Montoya, 2002). A key strength of this method lies in its universality, as it is based on the assumption that most organisms share a similar nitrogen metabolism, with trophic discrimination factor (TDF_Glx‐Phe_) of 7.6‰ (O'Connell, 2017). However, previous feeding experiments have shown that arachnids exhibit unusually low TDF_Glx‐Phe_ of 1.9‰ (Pollierer et al., 2019). Our data were compatible with the ^15^N‐fractionation in spiders from those feeding experiments. Considering their potential prey with the lowest Δ^15^N_Glx‐Phe_ (i.e., basal prey), spiders showed only very little ^15^N enrichment above basal (average 2.4‰), especially for blue sources (average −0.8‰). Therefore, our data can only be explained if the spiders were herbivorous, which we have ruled out, or if they generally have a lower TDF, which supports the laboratory‐derived spider TDF_Glx‐Phe_. Our results are also supported by recent studies attempting to estimate spider TP and finding unusually low TP, such as in a riparian food web (Wang et al., 2024, but see the potential confounding effect of source reliance, as discussed above), and in terrestrial food webs where spiders were found to have a TP of 2.2 using a non‐specific spider TDF_Glx‐Phe_ (Van Houtan et al., 2024). Thus, we are confident that the feeding experiments provided a TDF value applicable to wild conditions and consistent with expectations regarding spider trophic ecology. It is worth noting that using a different TDF in our study would have affected only the absolute TP estimates for spiders (and provided unrealistic results), but not the relative comparison between energy channels. This low spider TDF likely stems from specificities of spider external digestion and guanine nitrogen accumulation (see Pollierer et al., 2019, for more details).

An additional question involves web‐building spiders, as no feeding experiments have been conducted on this group. Protein excretion can alter amino acid metabolic flux and lower TDF, as seen in gastropods that release large amounts of protein‐rich saliva (Choi et al., 2018). Since web‐building spiders produce larger quantities of silk, made of amino acids (Craig et al., 2000), this raises questions about how silk production might impact TDF. In our study, comparing web‐building and hunting spiders was challenging, as they typically rely on different energy channels (e.g., all spiders of the green energy channel are web‐building ones). Despite this, we found that both spider types had similar TPs in blue and brown energy channels, with web‐building spiders showing only slightly (but not significantly) lower TPs. This suggests that web‐building likely has little to no effect on TDF, although further studies are needed for confirmation.

We also observed unexpectedly low TPs in ground beetles (family Carabidae). Shore predators (genus *Bembidion*) and leaf litter‐dwelling predators (genus *Limodromus*) had TPs of 2 and 2.6, respectively, lower than expected for predators (TP ≥ 3). Without controlled feeding experiments, it is difficult to determine whether this reflects an incorrect TDF and/or if it accurately represents omnivory in these ground beetles, as suggested by previous studies in *Bembidion* (Kulkarni et al., 2015).

All in all, we raise caution regarding the universal application of CSI‐AA for estimating TPs of terrestrial predators in food chains involving invertebrates. When using Glu as a unique trophic amino acid, spiders exhibit such low TDF that they become isotopically almost “invisible,” a term previously used to describe protist trophic links (Gutiérrez‐Rodríguez et al., 2014). For instance, a bird feeding directly on mayflies versus feeding on spiders that consume mayflies would show only a 0.25 TP difference using the universal TDF. However, spiders fractionate more other amino acids, such as leucine and threonine. Therefore, combining δ^15^N values from multiple amino acids offers a promising avenue for improving TP estimates in predators, and may even help distinguish specific trophic links (e.g., spiders appear to leave a unique amino acid fractionation pattern).

### Blue, green, and brown energy channels show distinct FCL from autotrophs to spiders

Many studies on spider trophic ecology using bulk isotopes rely on relative comparisons (Sanders et al., 2015; Zuev et al., 2020). In contrast, we demonstrate that employing CSI‐AA, while carefully considering dietary sources and TDFs (see above), enables robust estimates of spider TP, with reasonably constrained CIs. An additional major advantage of CSI‐AA over traditional bulk isotope analysis is its ability to trace trophic transfer all the way back to autotrophs, capturing otherwise overlooked trophic steps such as those mediated by microbes and fungi (Steffan et al., 2015).

Our modeling of spider TP from energy channel use highlights a strong correlation between those two variables, demonstrating how energy from distinct channels flows differently to riparian predators (Figure 5a). The lowest spider TP was observed in the blue energy channel (TP ≈ 2.9), reflecting a classic producer‐herbivore‐predator chain. This simple structure may reflect intrinsic features of stream food chains, where omnivory is generally less common (Thompson et al., 2007). Still, omnivory has been documented in stream consumers (Pringle & Hamazaki, 1998), including stonefly larvae (Miyasaka & Genkai‐Kato, 2009), confirmed by our study (stonefly, TP = 2.4 ± 0.3). Recent CSI‐AA insights have shown that macroinvertebrate biomass in streams typically has a low integrated TP (2.4 ± 0.2), indicating that most of the invertebrate biomass is composed of herbivores (Ishikawa et al., 2017, 2025). Emergence data showed that emerging insect biomass at our site in April to October was composed of mayflies (51%), stoneflies (31%), and chironomids (17%) (Kowarik et al., 2023), supporting that most blue resources for spiders have low TPs. Additionally, aquatic predators are generally larger than their prey, meaning that emerging predators, such as damselflies and dragonflies (Hirvonen & Ranta, 1996), are less likely to be preyed on by spiders (Murakami, 1983; Nentwig & Wissel, 1986). While our results may be applicable to other riparian spiders relying on blue energy channels, they cannot be generalized to larger riparian predators, or streams with a high abundance of predatory invertebrates.

In the green energy channel, we observed an elongation of approximately half a TP (TP ≈ 3.6), influenced by higher TP values in some spider prey, such as true bugs (Heteroptera) and grass flies. This pattern suggests significant omnivory among green consumers, commonly seen in terrestrial arthropods (Collier et al., 2002), including true bugs (Eubanks et al., 2003). The elevated TP of grass flies (Opomyzidae), however, is unexpected, as it is typically assumed to have a strictly herbivorous diet (Nartshuk, 2014). This discrepancy highlights the need for further molecular and isotopic studies on the dietary habits of grass flies. The observed prevalence of omnivory may be driven by the comparatively low nutrient quality of green resources, particularly limited nitrogen, which may have led consumers to adapt by feeding at higher trophic levels (Denno & Fagan, 2003).

Finally, we observed the highest spider TP (TP ≈ 4.1) in the brown energy channel. Here, most detritivores, except for one (crane fly larvae), exhibited intermediate TPs between primary and secondary consumers. These findings align with previous research showing that detritivore TP, such as that of springtails, fluctuates between ~2.6 and 3.2 depending on leaf litter compositions (Lux et al., 2024). The extended FCL in this channel reflects an added trophic level, where fungi and bacteria (decomposers) initiate energy transfer by metabolizing leaf litter, thereby making recalcitrant energy accessible to invertebrate detritivores (Junggebauer et al., 2025; Osono, 2006), such as springtails and woodlice (TP ~ 3), which serve as prey for spiders. This decomposition process, analogous to animal heterotrophy, shows similar ^15^N enrichment of Glu (Steffan et al., 2015). FCL elongation driven by decomposers has gained recognition through CSI‐AA in various taxa, including bees (Steffan et al., 2019), earthworms (Potapov, Tiunov, et al., 2019), squirrels (Pauli et al., 2019), and mammals (Manlick et al., 2023). Given the critical role of brown resources for food webs (Potapov, 2024), incorporating decomposer‐mediated trophic steps is essential for accurately estimating consumer TP and FCL across ecosystems.

### Does blue, green, and brown producer quality affect the efficiency of trophic transfer to spiders?

The drivers of FCL remain a debated topic in food web ecology leading to several hypotheses (Potapov, Brose, et al., 2019; Pringle & Hutchinson, 2020; Ward & McCann, 2017). FCL depends on the quantity of energy and nutrients produced at the base of the food web, as well as trophic transfer efficiency (i.e., the proportion of energy and nutrients passed from prey to consumers). However, disentangling the effects of these two factors on FCL is challenging (Mehner et al., 2022), making comparisons between ecosystems difficult. An alternative approach to studying changes in energy and nutrient flow is to examine the issue from a predator's perspective: the lower the predator's TP, the less energy is lost between trophic levels, indicating a more efficient transfer of basal energy up the food chain. This can serve as a proxy for what is referred to as “ecosystem efficiency” (Stukel et al., 2024), which reflects the number of trophic links and the amount of biomass lost between autotrophs and a given predator. Here, we adopted this approach by assessing through spiders' TPs how many trophic links are needed from primary producers to spiders (Figure 5b). Applied to riparian spiders, this method enables a comparison of ecosystem efficiency across blue, green, and brown energy channels.

The three energy channels differ significantly in their basal nutritional quality. The C:N ratios of stream biofilm (~15) (Romaní et al., 2004), grass (~30) (Sardans et al., 2012), and senescent leaves (~50) (Yuan & Chen, 2009) illustrate increasing protein content from brown to green to blue. Furthermore, other critical nutrients, such as PUFAs, are unevenly distributed among the basal resources but play essential metabolic roles in consumers (Twining et al., 2019). Long‐chain PUFAs like docosahexaenoic acid (DHA), which are vital for consumer health and function, are particularly abundant in blue resources (Hixson et al., 2015). In contrast, green resources provide lower levels of PUFAs, mainly short‐chain ones such as linoleic acid (Mathieu‐Resuge et al., 2022), while brown resources have minimal PUFA content (Ebm et al., 2021). Other molecules, such as lignin, are indigestible and make the assimilation of terrestrial resources recalcitrant to consumers, decreasing the nutritional value of green and brown resources (Brett et al., 2017). Altogether, the differences in macromolecular composition show increasing nutritional quality from brown to green to blue resources.

Our findings show that spider TP inversely correlates with nutrient quality, following the trend: blue > green > brown (Figure 5b), supporting that nutrient quality could affect ecosystem efficiency. In particular, spiders exhibited similar protein content across energy channels, and only slightly different total PUFA content within spider groups (variation in a 1:2 ratio) (Saboret, Drost, et al., 2024). The increase in the amount of trophic transfers may act as a compensatory mechanism for low nutrient amounts in basal resources, with intermediate trophic levels enhancing nutrient accumulation (Zhong et al., 2025). For example, bacteria and fungi at the base of the brown channel accumulate proteins and lipids (Swift et al., 1979), while detritivores like Collembola can accumulate long‐chain PUFAs (Chamberlain & Black, 2005; Pollierer et al., 2010). These findings suggest that variations in spider TP may arise from differences in ecosystem efficiency linked to the nutritional quality of basal resources. This finding can help to understand patterns of FCL, as we show that more trophic steps are needed to sustain spiders from terrestrial autotrophs than from aquatic ones, and so more energy and biomass are generally lost (Figure 5b). Nevertheless, in other ecosystems, food web specificities must be considered to link ecosystem efficiency to changes in FCL, including consumer trophic ecology (e.g., some species are more efficient than others), environmental effects on consumer trophic transfer efficiency (Dickman et al., 2008; Peace, 2015), and the potential impact of trophic cascades on trophic transfer efficiency (Peace, 2015), especially when new trophic levels are introduced (Carpenter et al., 2001; Terborgh & Estes, 2013).

### Considering changes in FCL in rewiring food webs

FCL is a crucial feature of food webs, as compounds can accumulate up food chains, a process commonly known as biomagnification (Kelly et al., 2007). This occurs for harmful substances, like mercury (Lavoie et al., 2013), but also for essential nutrients, such as long‐chain PUFAs, with key molecules like DHA being preferentially accumulated in consumers (Kainz et al., 2006; Persson & Vrede, 2006; Saboret, Drost, et al., 2024). Changes in FCL, therefore, could affect the landscape of nutrients and harmful compounds for consumers (Shipley et al., 2024).

Global change affects ecosystem productivity in various ways, resulting in shifts in how consumers utilise energy channels within a meta‐ecosystem, a phenomenon referred to as food web rewiring (Bartley et al., 2019). For example, warming‐induced increase in glacier meltwater reduces river production and, in turn, increases fish reliance on subsidies (Saboret, Moccetti, et al., 2024). Food web rewiring holds particularly true for riparian ecosystems (Larsen et al., 2016), in which land‐use change affects the export of blue resources (Ohler et al., 2024), warming induces a change in the timing of blue insect emergence (Shipley et al., 2022) and can also increase the relative importance of the brown energy channel (Manlick et al., 2024; Potapov et al., 2024).

Our study emphasizes the importance of accounting for shifts in predator TP due to food web rewiring. Specifically, the loss of blue energy channels and the increasing reliance on brown ones can significantly impact both the efficiency of biomass transfer and the bioaccumulation of various compounds, including potentially harmful substances.

## AUTHOR CONTRIBUTIONS

All authors developed the idea and conceptualized the study design. Carmen Kowarik collected the samples (2020), and Bastiaan J. W. Drost, Grégoire Saboret, and Maja Ilić collected the additional samples (2023). Bastiaan J. W. Drost and Carmen Kowarik identified invertebrates and prepared samples. Grégoire Saboret and Carsten J. Schubert analyzed fatty acids and amino acid isotopes. Grégoire Saboret analyzed the data and took the lead in writing the manuscript. All authors provided important feedback which helped shape the research and manuscript.

## CONFLICT OF INTEREST STATEMENT

The authors declare no conflicts of interest.

## Supporting information


Appendix S1.

## Data Availability

Data and code (Saboret, 2025) are available in the Open Science Framework repository at https://doi.org/10.17605/OSF.IO/5E6G8.
