# Supplementary material for: Reliance on blue, green, and brown energy channels drives a shift in the trophic position of riparian spiders
Source: Ecology. 2026 Jan 14;107(1):e70264. doi: 10.1002/ecy.70264 (PMC12803772; doi:10.1002/ecy.70264)
Supplement: Supplementary file 1 — Appendix S1. [file ECY-107-e70264-s001.pdf]

Appendix S1

Reliance on blue, green, and brown energy channels drives a shift in the trophic position of riparian spiders

Grégoire Saboret, Bastiaan J. W. Drost, Carmen Kowarik, Maja Ilić, Martin M. Gossner, and Carsten J. Schubert

Ecology

Section S1

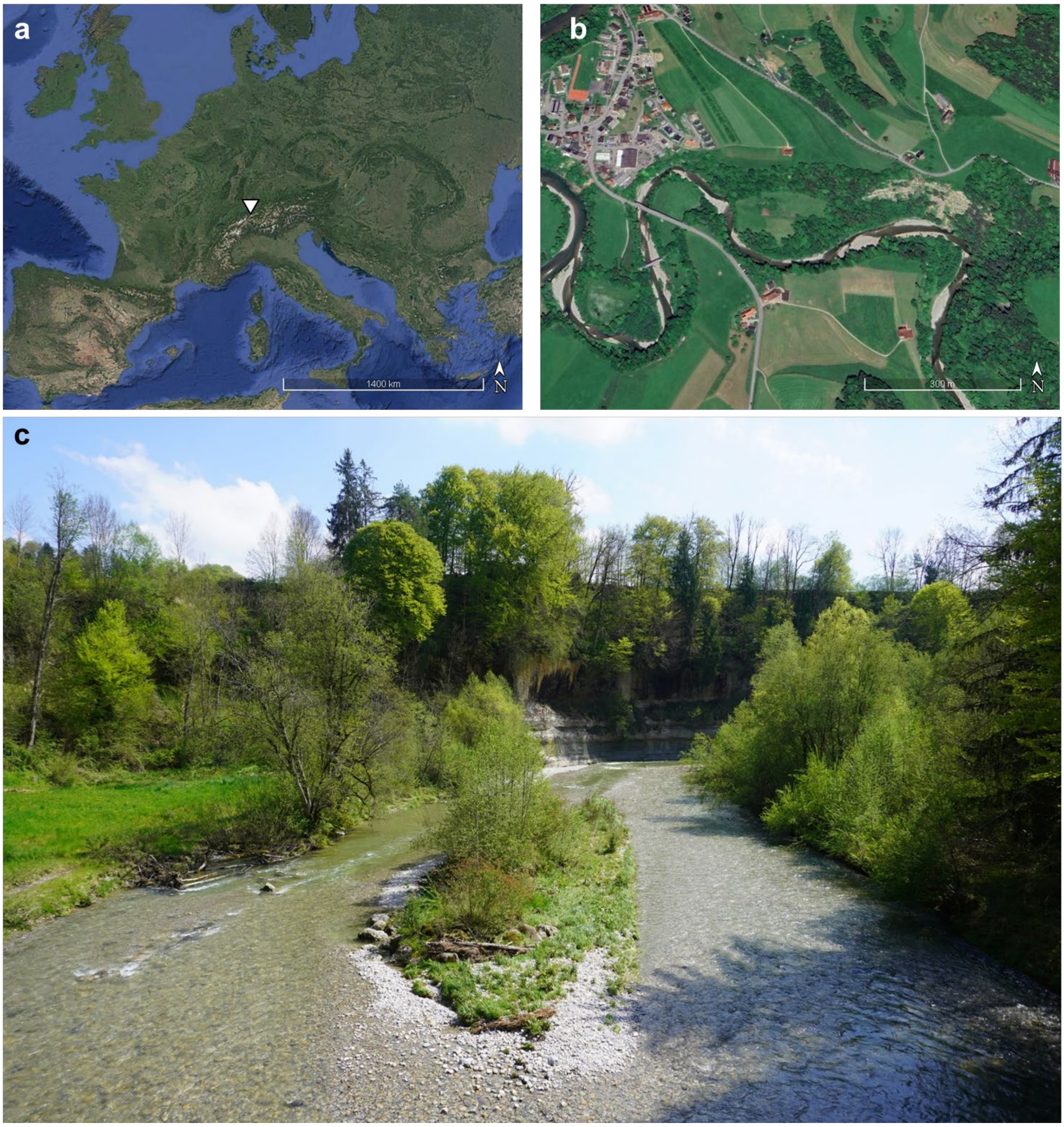

Figure S1. Study site.

(a) Location of the study site in Europe (triangle). **(b)** Aerial view of the study site, showing the River Necker bordered by forests, meadows, and agricultural fields. Satellite imagery obtained from Google Earth™ (© Google Earth; image © SIO, NOAA, U.S. Navy, NGA, GEBCO, Landsat/Copernicus). **(c)** Photograph of the study site, taken from the river side. Photo credit for (c): Maja Ilić.

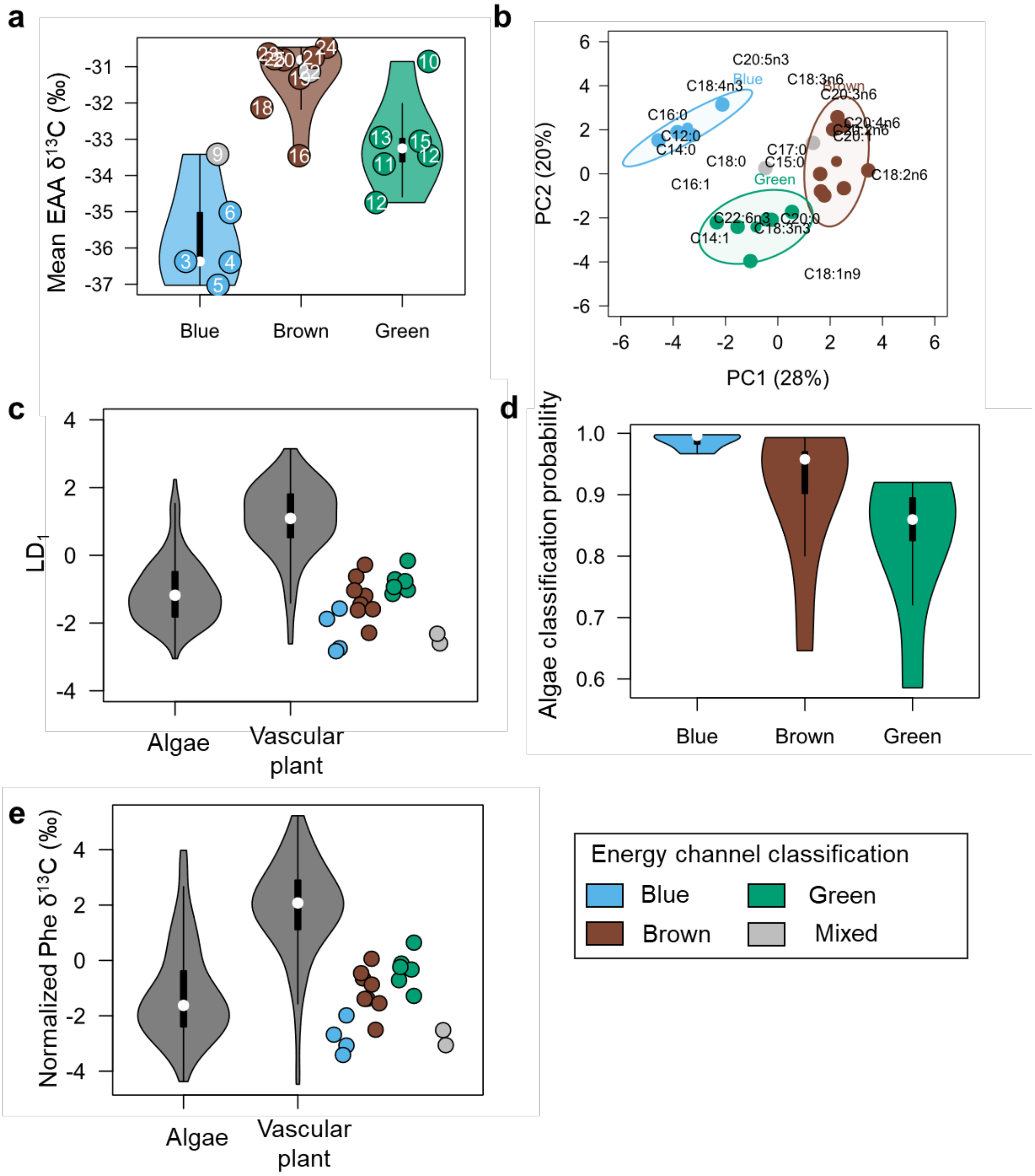

Figure S2. Evaluation of the importance of algae in supporting the blue energy channel **(a)** Violin plot of the mean δ^{13}C values of four essential amino acids (Phe, Leu, Ile, and Val) among the three groups. Numbers refer to sample identifications in Saboret (2025) (see https://osf.io/5e6g8/files/, Invertebrate_AA_isotope_data.xlsx) **(b)** Principal component analysis of fatty acid relative composition. Dots represent individuals, and ellipses show the 80% distribution for the three categories. Fatty acids indicate their relative contribution to the axes. **(c)** Violin plot of the linear discriminant analysis (LDA) axis based on the fingerprinting of δ^{13}C values from four essential amino acids (Phe, Leu, Ile, and Val) and relevant literature

values for algae and vascular plants (Section S2). **(d)** Probability distribution of classification as aquatic based on the LDA shown in (c). **(e)** Violin plot of normalized Phe $\delta^{13}C$ values (i.e., relative difference between Phe and the mean essential amino acid value, shown in b), and relevant literature values for algae and vascular plants. Dots represent predictions for invertebrates across the four categories, including the mixed category (grey).

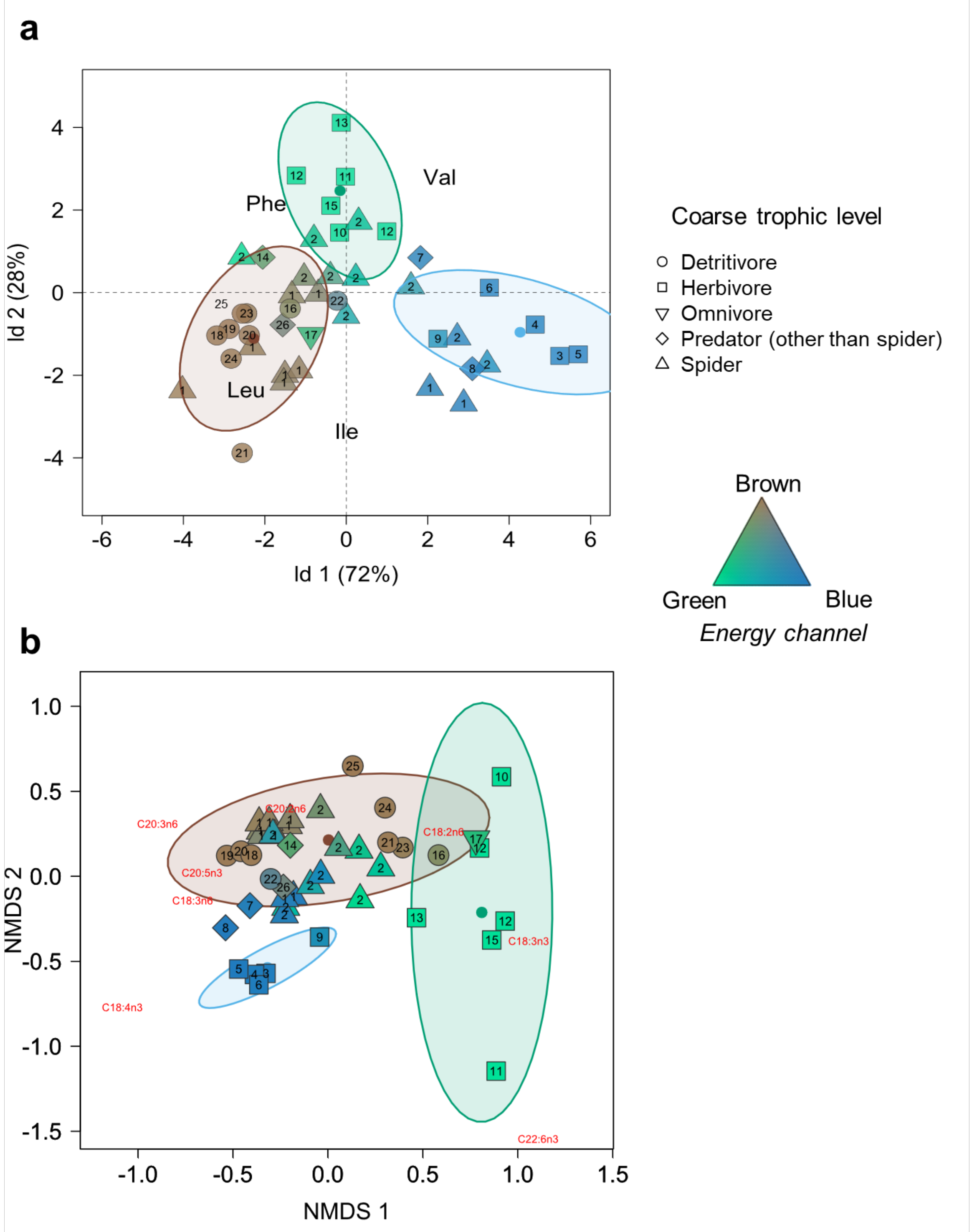

Figure S3. Characterization of energy channel (blue, green, brown) reliance.
(a) Linear Discriminant Analysis (LDA) plot of $\delta^{13}C$ values of four essential amino acids (Ile, Leu, Phe, and Val), using herbivores and detritivores as source end-members. Ellipses show the 80% distribution of the sources. Adapted from Saboret et al. (2024). **(b)** Non-metric Multidimensional Scaling (NMDS) plot of the relative content of eight polyunsaturated fatty

acids (PUFAs) based on Bray-Curtis distance. Red text indicates PUFA contributions to the NMDS plot. Ellipses show the 80% distribution of the sources. (a, b) Each dot represents a sample, numbers refer to species codes in Saboret (2025) (see https://osf.io/5e6g8/files/, Invertebrate_AA_isotope_data.xlsx), and colors indicate energy channel use estimates (see legend).

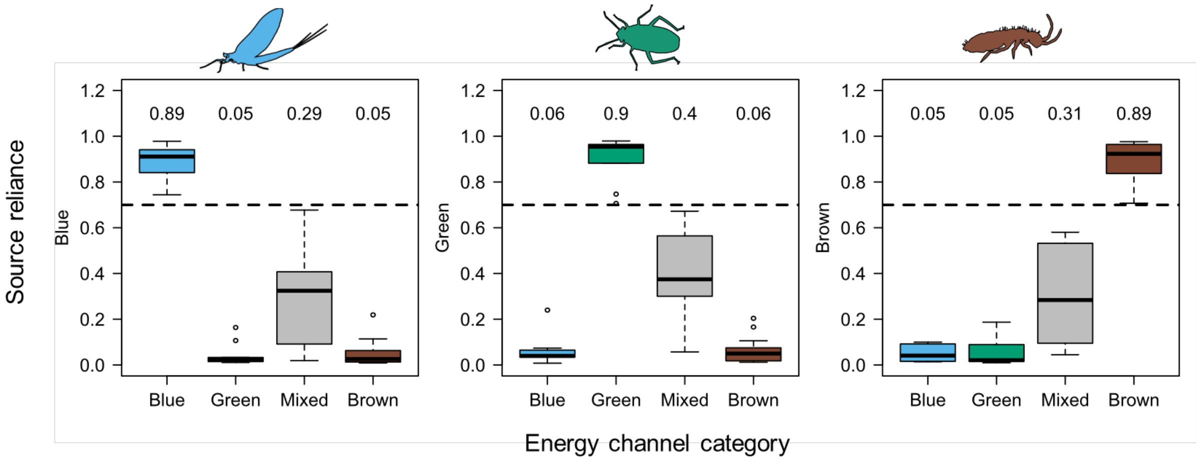

Figure S4. Energy channel categories

Relative distribution of the three source reliance (from left to right: Blue, Green, and Brown) across the four energy channel categories. Numbers on top represent the group mean by source. The dashed line indicates the threshold (70%) at which consumers are considered source specialists. The 'mixed' category includes all consumers with no reliance above 70%. Figure created with species silhouettes sourced from Phylopic.org, licensed under a CC0 1.0 Universal Public Domain Dedication license.

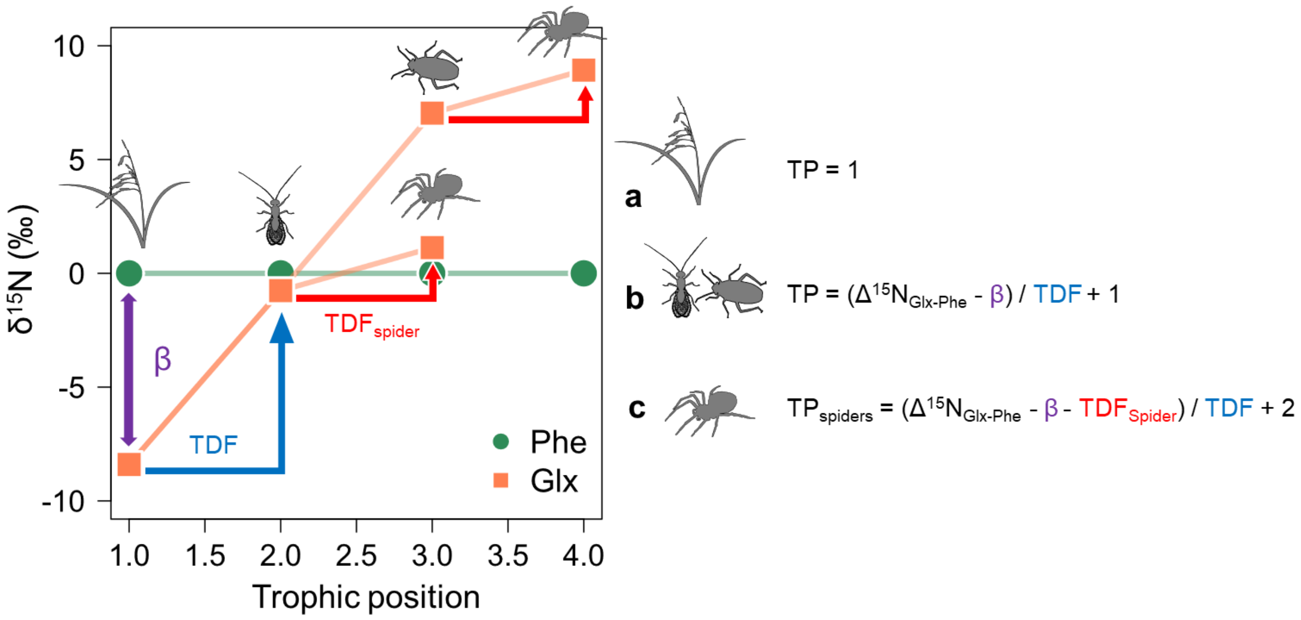

Figure S5. Equations for trophic position estimates

The graph shows two hypothetical green food chains with associated Phenylalanine (Phe, green circles) and Glutamic acid + glutamine (Glx, orange squares) $\delta^{15}N$. X-axis shows trophic position (TP). **(a)** At TP = 1, primary producers fractionate Glx over Phe (β value). In this simplified example, none of the consumers fractionate Phe, which is set to 0. **(b)** Consumers fractionate Glx by one TDF. Their TP is calculated by estimating the number of trophic steps (TDF), using the measured $\Delta^{15}N_{Glx\text{-}Phe}$, subtracted for primary producer fractionation (- β), which is set as TP = 1. **(c)** As spiders show unusual TDF, their trophic position is estimated by using equation (b), but constraining the last trophic step as being the spider. This results in subtracting TDF_{spider}, setting TP = 2, and measuring the additional contribution to TP by intermediate consumers. Figure made with species silhouettes sourced from Phylopic.org under CC0 1.0 Universal licensing. Authors listed in Saboret (2025) (see https://osf.io/5e6g8/files/, Figure_credits.xlsx).

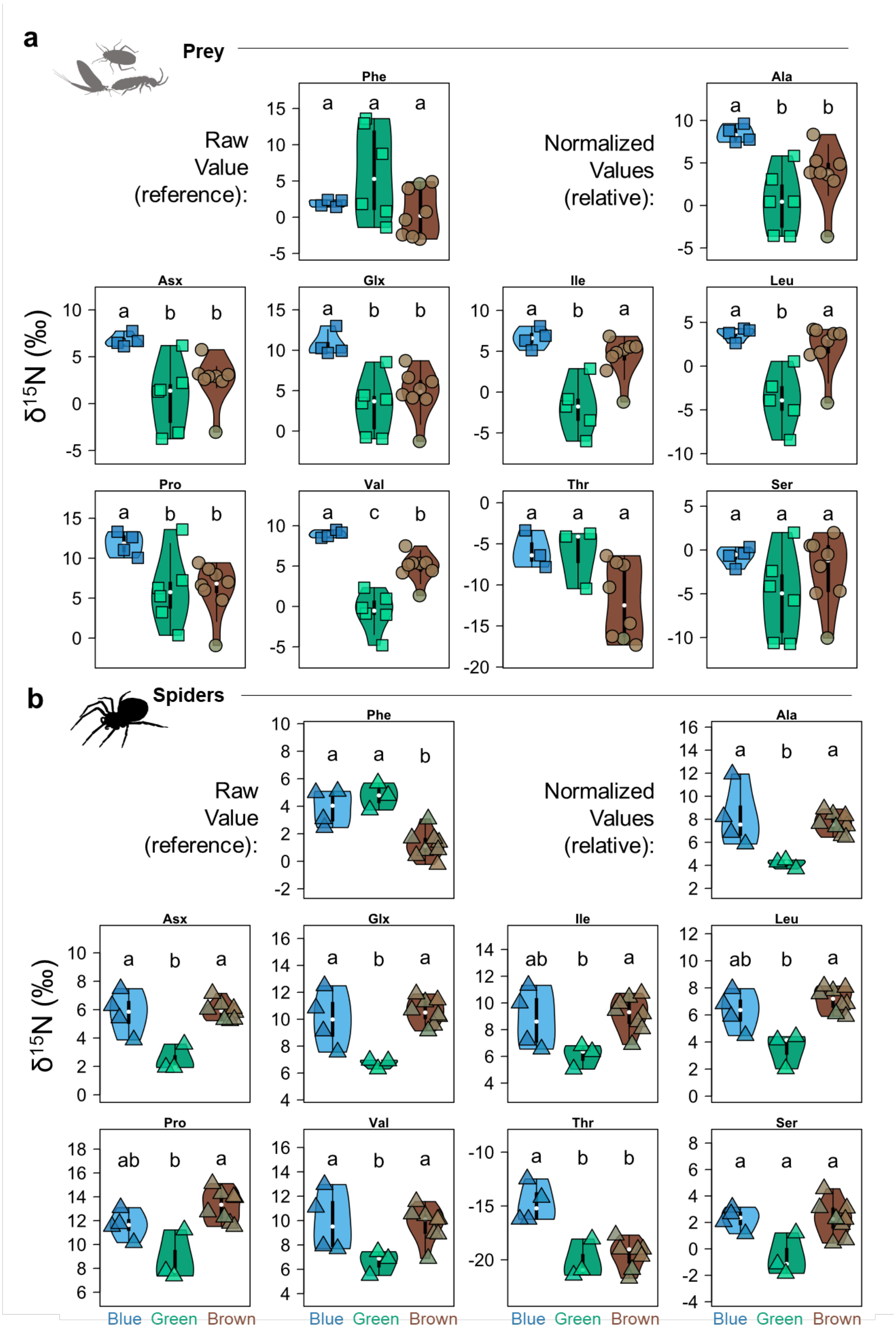

Figure S6. $\delta^{15}N_{AA}$ in spiders and their prey (herbivores/detritivores). Violin plots showing normalized $\delta^{15}N$ amino acids in three different energy channels. All $\delta^{15}N_{AA}$ are normalized (i.e, the value is relative to Phe value), except for Phe (raw value). The top panel **(a)** represents prey, and the bottom panel **(b)** represents spiders. Letters indicate significant differences

between groups ($P<0.05$, Kruskal-Wallis test with post-hoc comparisons using the Fisher's least significant difference with Holm-Bonferonni corrections). Figure created with species silhouettes sourced from Phylopic.org, licensed under a CC0 1.0 Universal Public Domain Dedication license.

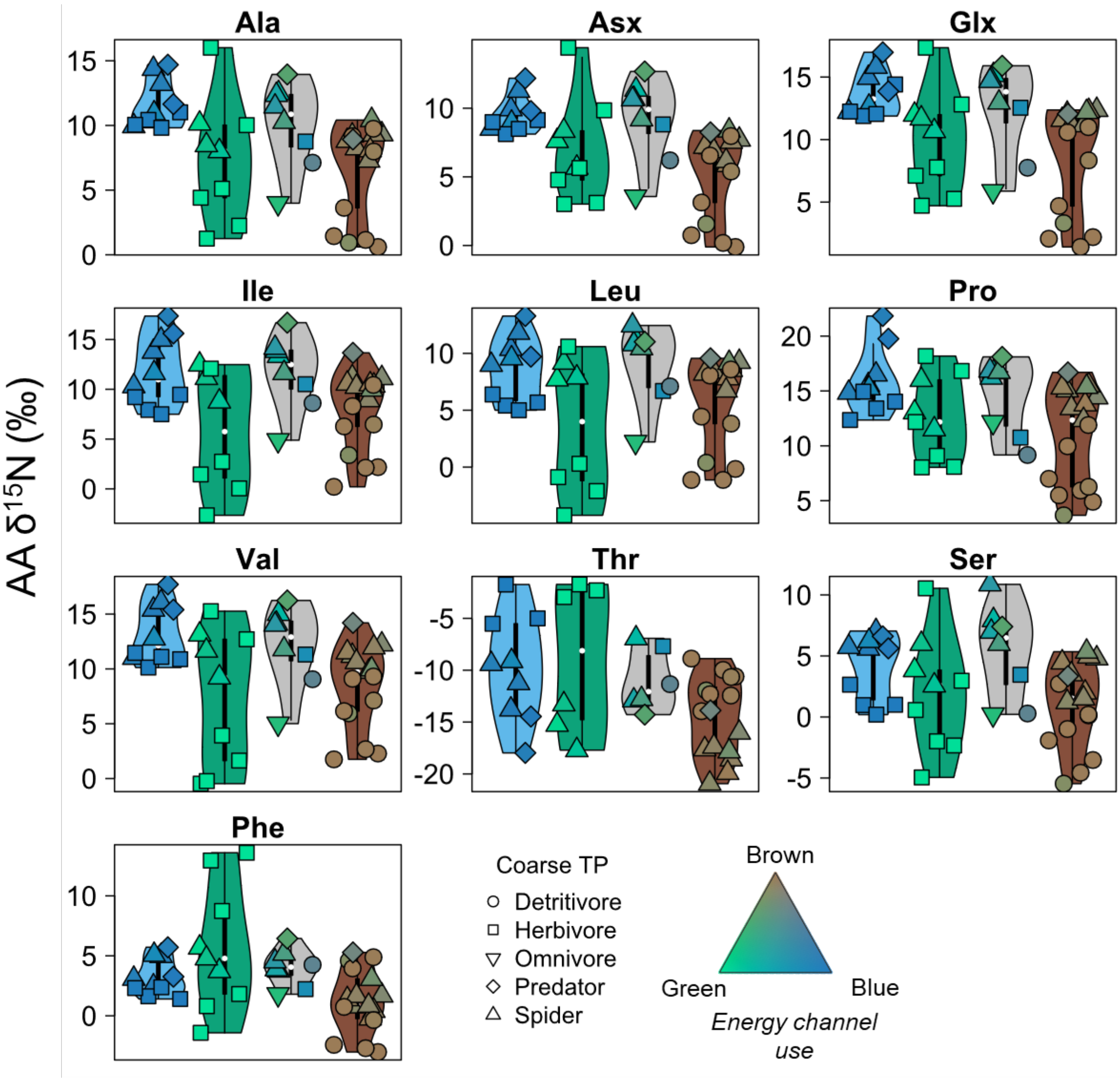

Figure S7. $\delta^{15}N_{AA}$ for different energy channels. Violin plots showing $\delta^{15}N$ amino acids in the four different food chains, from left to right: blue, green, mixed and brown. Symbol shapes and colors show sample category and source reliance. Letters indicate significant differences between groups ($P < 0.05$, Kruskal-Wallis test with post-hoc comparisons using the Fisher's least significant difference with Holm-Bonferonni corrections).

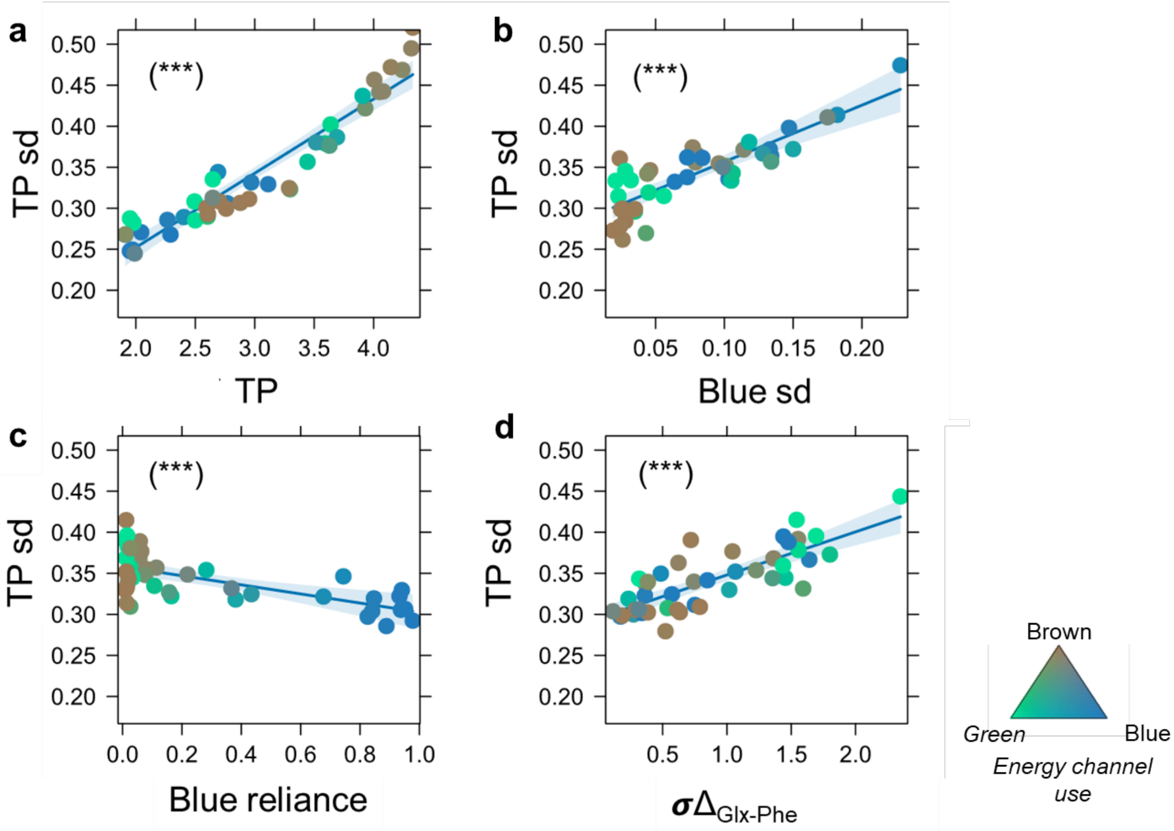

Figure S8. Uncertainty in trophic position estimates. Linear relationships between trophic position uncertainty (as one standard deviation, y-axis) and mean trophic position estimates (a), uncertainty in aquatic reliance (bs), blue reliance (c), and uncertainty in analytical measurement of $\Delta^{15}N_{Glx\text{-}Phe}$ ($\boldsymbol{\sigma}_{\Delta Glx\text{-}Phe}$). The plots display partial residuals, with circles representing samples, and dot color indicating energy channel use (legend).

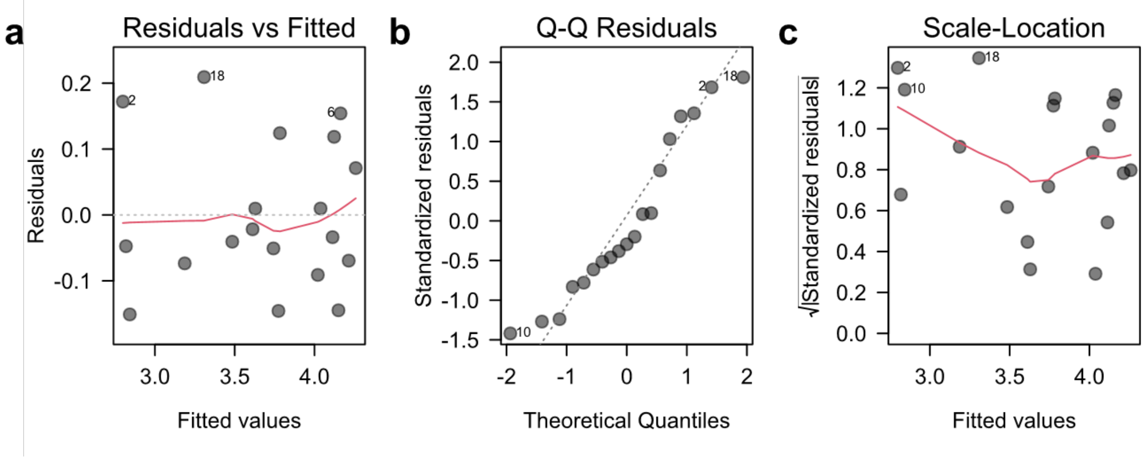

Figure S9. Diagnostic plot of linear regression of TP against energy channel use. **(a)** Linearity: Residuals vs. TP. **(b)** Normality of residuals: Q-Q plot of residuals. **(c)** Homogeneity of variance: Spread-location plot.

Section S2

Characterization of the relative importance of algae in supporting the blue energy channel

Our emerging insect samples were primarily composed of mayflies (51% of biomass), which are generally considered grazers of autochthonous (algal) production, thus representing a classic “blue” energy channel. However, allochthonous organic carbon can be an important nutrient source for aquatic consumers, particularly in stream ecosystems in the form of leaf litter (Wallace et al. 1997, Brett et al. 2017, Gounand et al. 2018). Here, we investigate whether primary consumers within this blue energy channel derive their nutrients predominantly from autochthonous or allochthonous sources. Distinguishing between these sources at the primary consumer level can be challenging. For instance, bulk stable isotope tracers have limitations (Ishikawa et al. 2020), mainly because of isotopic composition overlaps (Finlay 2004), and spatio-temporal variation of benthic stream production (Richardson et al. 2009), as highlighted by seasonal invertebrate bulk carbon isotopes at our site (Kowarik et al. 2021). Moreover, terrestrial leaf litter is not always assimilated directly: invertebrates feeding on leaves may in fact consume biofilms growing on the leaf surface, including algae (Guo et al. 2016b), a phenomenon known as the "peanut butter hypothesis" (Cummins 1974). To address these complexities, we employed molecular biomarkers (fatty acids and essential amino acids) to assess whether our data support a predominant reliance on autochthonous resources. Data for this analysis are published in Saboret et al. (2024). Our aim was not to produce a quantitative estimate (beyond the scope of the present study), but rather to evaluate the plausibility of our assumption regarding the blue reliance of emerging insects.

Fatty acids

Some essential fatty acids, such as polyunsaturated fatty acids (PUFAs), are synthesized exclusively by primary producers, and algae and terrestrial plants exhibit distinct fatty acid profiles (Twining et al. 2016). Specifically, algae are rich in omega-3 fatty acids, such as eicosapentaenoic acid (EPA, 20:5n3) and stearidonic acid (SDA, 18:4n3). In contrast, terrestrial plants primarily contain short-chain omega-6 fatty acids, including linoleic acid (LIN, 18:2n6) (Hixson et al. 2015). These fatty acids can serve as biomarkers, though their

direct quantification is challenging due to substantial modifications in relative abundance by primary consumers (Dalsgaard et al. 2003, Galloway and Budge 2020).

To evaluate whether fatty acid patterns were consistent with an autochthonous nutrient source in blue primary herbivores, we conducted a principal component analysis (PCA) on relative fatty acid composition. The results showed that blue consumers clustered together and were particularly characterized by higher levels of EPA and SDA, both known aquatic biomarkers (Figure S1b). Notably, SDA was below detection limits in all green primary consumers. In contrast, blue herbivores exhibited low levels of LIN (average 4%), a terrestrial marker that was more abundant in green (9%) and brown (15%) energy channels.
The ratio of omega-3 to omega-6 fatty acids can also serve as an indicator of algal reliance, with a ratio >1 typically reflecting dominance of algal-derived nutrition in stream invertebrates (Guo et al. 2016a). Here, blue herbivores exhibited high omega-3/omega-6 ratios (>2, mean: 3, max: 6), with the exception of a single stonefly sample (ratio = 1), which was further classified as part of a mixed energy channel (see main text: *Methods, Energy channel use*).
Altogether, the fatty acid profiles of blue herbivores were consistent with strong algal dominance, supporting the interpretation that these consumers rely primarily on autochthonous resources (algae).

Essential amino acid carbon isotopes

Essential amino acids (EAAs) serve as robust dietary tracers because they are only synthesized by primary producers and are transferred up the food chain with little to no carbon isotopic fractionation (Manlick and Newsome 2022). Blue primary consumers had a distinct EAA $\delta^{13}C$ signature, with an overall depletion of ~3‰ compared to green and brown primary consumers (Figure S1a), suggesting distinct origin of EAAs.

Additionally, primary producers exhibit characteristic offsets in EAA $\delta^{13}C$ values, a pattern known as EAA fingerprinting (Larsen et al. 2009), which is preserved up to consumers (Whiteman et al. 2019, Manlick and Newsome 2022). This fingerprinting arises primarily from isotopic fractionation during anabolic processes in primary producers and is largely independent of local baseline variation or environmental growth conditions (Larsen et al. 2013, Elliott Smith et al. 2022, Stahl et al. 2023). Previous studies have demonstrated that EAA fingerprinting can effectively distinguish between aquatic and terrestrial reliance in consumers (Thorp and Bowes 2017, Liew et al. 2019, Arsenault et al. 2022, Saboret et al. 2023).

Here, we used the fingerprinting approach to explore wherever the blue herbivore fingerprinting was consistent with dominant reliance on algae. We excluded a quantitative model because of the lack of local baselines (Vane et al. 2025), and the lack of threonine values in our dataset. We incorporated literature data from Arsenault et al. (2022), filtering plant species to only those relevant to our study sites (e.g., excluding C4 plants and cacti). We then performed a linear discriminant analysis (LDA) using four measured EAAs (isoleucine, leucine, phenylalanine and valine) to differentiate between algae and vascular plants (Figure S1c). This classification was primarily driven by phenylalanine (Phe) and leucine (Leu) (loadings of 0.34 and -0.51, respectively). We applied the LDA to consumers, and found that blue herbivores had an algal pattern (Figure S1c,e), and a high probability of classification (>97%) as aquatic (Figure S1d). The misclassification of brown detritivores likely reflects the absence of appropriate end-members (fungi and bacteria) in the model. Similarly, uncertainties in the classification of green herbivores may stem from model limitations due to the lack of local isotopic baselines (Vane et al. 2025). While the approach is not quantitative, EAA fingerprinting of blue herbivores was consistent with a strong reliance on algal resources. In particular, Phe $\delta^{13}C$ values, which tend to be relatively enriched in vascular plant sources (Saboret et al. 2023), were notably depleted in blue herbivores (Figure S1e), comforting a dominant contribution from algal production.

Conclusion

Both the relative abundance of fatty acids and the carbon isotopic composition of essential amino acids exhibited patterns characteristic of algal production. These biomarkers support the assumption of a dominant reliance on algal resources by blue herbivores.

References

Arsenault, E. R., J. H. Thorp, M. J. Polito, M. Minder, W. K. Dodds, F. Tromboni, A. Maasri, M. Pyron, B. Mendsaikhan, A. Otgonganbat, S. Altangerel, S. Chandra, R. Shields, C. Artz, and H. Bennadji. 2022. Intercontinental analysis of temperate steppe stream food webs reveals consistent autochthonous support of fishes. Ecology Letters:ele.14113.

Brett, M. T., S. E. Bunn, S. Chandra, A. W. E. Galloway, F. Guo, M. J. Kainz, P. Kankaala, D. C. P. Lau, T. P. Moulton, M. E. Power, J. B. Rasmussen, S. J. Taipale, J. H. Thorp, and J. D. Wehr. 2017. How important are terrestrial organic carbon inputs for secondary production in freshwater ecosystems? Freshwater Biology 62:833–853.

Cummins, K. W. 1974. Structure and function of stream ecosystems. BioScience 24:631–641.

Dalsgaard, J., M. St. John, G. Kattner, D. Müller-Navarra, and W. Hagen. 2003. Fatty acid trophic markers in the pelagic marine environment. Pages 225–340 Advances in Marine Biology. Elsevier.

Elliott Smith, E. A., M. D. Fox, M. L. Fogel, and S. D. Newsome. 2022. Amino acid δ^{13}C fingerprints of nearshore marine autotrophs are consistent across broad spatiotemporal scales: An intercontinental isotopic dataset and likely biochemical drivers. Functional Ecology:1365-2435.14017.

Finlay, J. C. 2004. Patterns and controls of lotic algal stable carbon isotope ratios. Limnology and Oceanography 49:850–861.

Galloway, A. W. E., and S. M. Budge. 2020. The critical importance of experimentation in biomarker-based trophic ecology. Philosophical Transactions of the Royal Society B: Biological Sciences 375:20190638.

Gounand, I., C. J. Little, E. Harvey, and F. Altermatt. 2018. Cross-ecosystem carbon flows connecting ecosystems worldwide. Nature Communications 9:4825.

Guo, F., M. J. Kainz, F. Sheldon, and S. E. Bunn. 2016a. The importance of high-quality algal food sources in stream food webs–current status and future perspectives. Freshwater Biology 61:815–831.

Guo, F., M. J. Kainz, D. Valdez, F. Sheldon, and S. E. Bunn. 2016b. High-quality algae attached to leaf litter boost invertebrate shredder growth. Freshwater Science 35:1213–1221.

Hixson, S. M., B. Sharma, M. J. Kainz, A. Wacker, and M. T. Arts. 2015. Production, distribution, and abundance of long-chain omega-3 polyunsaturated fatty acids: a fundamental dichotomy between freshwater and terrestrial ecosystems. Environmental Reviews 23:414–424.

Ishikawa, N. F., J. C. Finlay, H. Uno, N. O. Ogawa, N. Ohkouchi, I. Tayasu, and M. E. Power. 2020. Combined use of radiocarbon and stable carbon isotopes for the source mixing model in a stream food web. Limnology and Oceanography 65:2688–2696.

Kowarik, C., D. Martin-Creuzburg, and C. T. Robinson. 2021. Cross-Ecosystem Linkages: Transfer of Polyunsaturated Fatty Acids From Streams to Riparian Spiders via Emergent Insects. Frontiers in Ecology and Evolution 9:707570.

Larsen, T., D. L. Taylor, M. B. Leigh, and D. M. O'Brien. 2009. Stable isotope fingerprinting: a novel method for identifying plant, fungal, or bacterial origins of amino acids. Ecology 90:3526–3535.

Larsen, T., M. Ventura, N. Andersen, D. M. O'Brien, U. Piatkowski, and M. D. McCarthy. 2013. Tracing Carbon Sources through Aquatic and Terrestrial Food Webs Using Amino Acid Stable Isotope Fingerprinting. PLoS ONE 8:e73441.

Liew, J. H., K. W. J. Chua, E. R. Arsenault, J. H. Thorp, A. Suvarnaraksha, A. Amirrudin, and D. C. J. Yeo. 2019. Quantifying terrestrial carbon in freshwater food webs using

amino acid isotope analysis: Case study with an endemic cavefish. Methods in Ecology and Evolution 10:1594–1605.

Manlick, P. J., and S. D. Newsome. 2022. Stable isotope fingerprinting traces essential amino acid assimilation and multichannel feeding in a vertebrate consumer. Methods in Ecology and Evolution:2041–210X.13903.

Richardson, D. C., L. A. Kaplan, J. Denis Newbold, and A. K. Aufdenkampe. 2009. Temporal dynamics of seston: A recurring nighttime peak and seasonal shifts in composition in a stream ecosystem. Limnology and Oceanography 54:344–354.

Saboret, G. 2025. Spider Trophic Position - Amino Acid Stable Isotopes. Open Science Framework. https://doi.org/10.17605/OSF.IO/5E6G8.

Saboret, G., B. J. W. Drost, C. Kowarik, C. J. Schubert, M. M. Gossner, and M. Ilić. 2024. Quantifying the utilisation of blue, green and brown resources by riparian predators: A combined use of amino acid isotopes and fatty acids. Methods in Ecology and Evolution:2041–210X.14371.

Saboret, G., D. Stalder, B. Matthews, J. Brodersen, and C. J. Schubert. 2023. Autochthonous production sustains food webs in large perialpine lakes, independent of trophic status: Evidence from amino acid stable isotopes. Freshwater Biology 68:870–887.

Stahl, A. R., T. A. Rynearson, and K. W. McMahon. 2023. Amino acid carbon isotope fingerprints are unique among eukaryotic microalgal taxonomic groups. Limnology and Oceanography:lno.12350.

Thorp, J. H., and R. E. Bowes. 2017. Carbon Sources in Riverine Food Webs: New Evidence from Amino Acid Isotope Techniques. Ecosystems 20:1029–1041.

Twining, C. W., J. T. Brenna, N. G. Hairston, and A. S. Flecker. 2016. Highly unsaturated fatty acids in nature: what we know and what we need to learn. Oikos 125:749–760.

Vane, K., M. R. D. Cobain, and T. Larsen. 2025. The power and pitfalls of amino acid carbon stable isotopes for tracing origin and use of basal resources in food webs. Ecological Monographs 95:e1647.

Wallace, J. B., S. L. Eggert, J. L. Meyer, and J. R. Webster. 1997. Multiple Trophic Levels of a Forest Stream Linked to Terrestrial Litter Inputs. Science 277:102–104.

Whiteman, J., E. Elliott Smith, A. Besser, and S. Newsome. 2019. A Guide to Using Compound-Specific Stable Isotope Analysis to Study the Fates of Molecules in Organisms and Ecosystems. Diversity 11:8.
